# Recycling and Reuse of Spent LIBs: Technological Advances and Future Directions

**DOI:** 10.3390/molecules29133161

**Published:** 2024-07-02

**Authors:** Long Lv, Siqi Zhou, Changqi Liu, Yuan Sun, Jubing Zhang, Changsheng Bu, Junguang Meng, Yaji Huang

**Affiliations:** 1 School of Energy and Mechanical Engineering, Nanjing Normal University, Nanjing 210046, China; wangyi13172718632@163.com (L.L.); jubingzhang@njnu.edu.cn (J.Z.); csbu@njnu.edu.cn (C.B.); mengjunguang@njnu.edu.cn (J.M.); 2 State Key Laboratory of NBC Protection for Civilian, Beijing 100083, China; 3 Key Laboratory of Energy Thermal Conversion and Control of Ministry of Education, School of Energy and Environment, Southeast University, Nanjing 210096, China

**Keywords:** solid waste treatment, spent lithium-ion batteries, cathode material recycling, valuable metal extraction, cathode regeneration

## Abstract

Recovering valuable metals from spent lithium-ion batteries (LIBs), a kind of solid waste with high pollution and high-value potential, is very important. In recent years, the extraction of valuable metals from the cathodes of spent LIBs and cathode regeneration technology are still rapidly developing (such as flash Joule heating technology to regenerate cathodes). This review summarized the studies published in the recent ten years to catch the rapid pace of development in this field. The development, structure, and working principle of LIBs were firstly introduced. Subsequently, the recent developments in mechanisms and processes of pyrometallurgy and hydrometallurgy for extracting valuable metals and cathode regeneration were summarized. The commonly used processes, products, and efficiencies for the recycling of nickel–cobalt–manganese cathodes (NCM/LCO/LMO/NCA) and lithium iron phosphate (LFP) cathodes were analyzed and compared. Compared with pyrometallurgy and hydrometallurgy, the regeneration method was a method with a higher resource utilization rate, which has more industrial application prospects. Finally, this paper pointed out the shortcomings of the current research and put forward some suggestions for the recovery and reuse of spent lithium-ion battery cathodes in the future.

## 1. Introduction

Traditional alkaline batteries, nickel–cadmium batteries, nickel–metal hydride batteries, and lead-acid batteries can no longer meet society’s increasing energy storage demands due to factors such as low energy density and high self-discharge rates. To better understand the advantages of lithium-ion batteries compared to other types of batteries, Table 1 summarizes the key characteristics and recent developments of traditional alkaline batteries, nickel–cadmium batteries, nickel–metal hydride batteries, lead-acid batteries, and lithium-ion batteries. Lithium-ion battery technology plays an important role in modern society and is prevalent in various fields including mobile devices, electric transportation, renewable energy storage, portable medical devices, information technology, and communication systems. Its high energy density and lightweight characteristics make mobile devices more portable while providing reliable power sources for electric vehicles, reducing reliance on fossil fuels. In the field of renewable energy, lithium-ion batteries serve as efficient energy storage devices, balancing the supply and demand of renewable energy sources such as solar and wind power. The non-aqueous 3 V lithium-ion primary battery was first introduced to the market in 1969 [1]. In the late 1970s, Armand [2] was the first to propose the concept of a battery that enables the reversible movement of lithium ions between the positive and negative electrodes, utilizing intercalation materials with different potentials for each electrode. Since the lithium ions flowed back and forth between the two electrodes, this type of battery was commonly referred to as a rocking chair battery. Lazzari and Scroati quickly realized Armand’s idea by using tungsten dioxide and titanium disulfide as electrodes, completing multiple charge–discharge cycles under low voltage [3], and evaluating the battery characteristics of different active electrodes, emphasizing the importance of selecting suitable electrode materials [4]. At the same time, Goodenough et al. [5] proposed that LiCoO_2_ with good stability could be used as the new cathode for LIBs. Due to its excellent electrochemical performance, it remains the most widely used positive electrode for commercial LIBs today.

**Figure 1:**
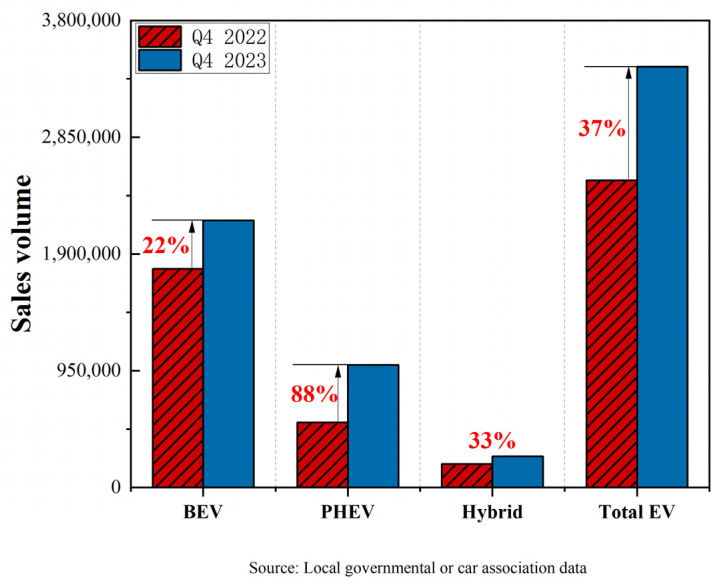
Comparison of electric vehicle sales between the fourth quarter of 2022 and the fourth quarter of 2023.

The commercialization of secondary LIBs can be traced back to 1991 when the Japanese company Sony introduced the LiCoO_2_/graphite anode LIB system and applied it to its Handycam camcorders, successfully providing reliable power for portable video recording devices [11]. This innovation marked the transition of lithium-ion battery technology from the laboratory to the commercial market, leading to significant breakthroughs in the field of mobile electronic devices [12,13]. Sony’s commercial lithium-ion batteries revolutionized the battery market, replacing some traditional rechargeable batteries and becoming the mainstream power source for mobile devices, digital cameras, and other electronic products. They also established the dominant position of LIBs in modern electronic products, promoting the continuous progress and development of lithium-ion battery technology. In 1997, General Motors’ EV1 electric vehicle became the first to adopt lithium-ion batteries as a power source [14], marking an innovative step in the application of LIB technology in the electric vehicle field, despite the electric vehicle market being in its early stages at that time. With increasing attention to clean energy and sustainable transportation, the use of LIBs in electric vehicles has gradually increased, becoming one of the key technologies driving the development of electric transportation. The application of LIBs in electric vehicles and other transportation means continues to expand, making significant contributions to sustainable transportation and environmentally friendly travel. In 2008, the Tesla Roadster was first delivered, becoming the first electric vehicle on the market to use lithium-ion batteries [15]. Subsequently, many car manufacturers entered the electric vehicle field, with Tesla continuing to introduce more models, while other mainstream car manufacturers gradually entered the electric vehicle market. Starting in 2017, the global electric vehicle market has experienced explosive growth, with many countries introducing policies and regulations to promote the development of electric vehicles. Several automobile manufacturers have announced gradual shifts towards electrification. The sales figures for the top ten BEV (Battery Electric Vehicle) manufacturers worldwide from 2022 to 2023 are shown in Figure 1. Analysis based on Figure 1 shows a significant increase in electric vehicle (EV) sales in the fourth quarter of 2023 compared to those in the fourth quarter of 2022, including sales of plug-in hybrid electric vehicles (PHEVs) and battery electric vehicles (BEVs), respectively. This growth trend directly drives a rapid increase in the demand for LIBs since LIBs are the core power source for these electric vehicles. As the electric vehicle market continues to expand, the production and supply chain of LIBs face significant pressure to meet the increasing demand [16].

According to the simulation results of Kapustin et al. [17], by 2040, the number of electric vehicles is projected to increase by 60–70 times compared to 2016, making up 12–28% of the total global vehicles. In 2019, the global production of lithium-ion batteries was approximately 200 GWh, and it is expected that the demand for lithium-ion batteries worldwide will increase nearly tenfold by 2030 [18]. With the continuous increase in the scale of production, sales, and application of power batteries, the problem of battery replacement and recycling after long-term use has become particularly prominent [19]. Lithium iron phosphate batteries and ternary lithium-ion batteries are two commonly utilized battery types in electric vehicles. For lithium iron phosphate batteries, they are generally considered unsuitable for use in electric vehicles when their capacity drops below 80% of the initial capacity. Ternary LIBs, on the other hand, can only be used for about 6 years after being charged approximately 2000 times.

Improper disposal of solid waste can lead to the release of harmful elements into the environment, which can affect human health and destabilize ecosystems, causing long-term environmental damage [20,21,22]. With the rapid growth in lithium battery production and the extensive adoption of electric vehicles, mobile devices, renewable energy, and other fields, the number of discarded lithium-ion batteries is also increasing. Improperly disposed of lithium-ion batteries may release harmful substances, polluting soil and water sources and jeopardizing the stability of ecosystems [23]. Effective recycling can minimize environmental pollution, protect the sustainable development of the ecological environment, and recover valuable and rare resources such as lithium, cobalt, nickel, and others contained within discarded lithium-ion batteries. This article reviews the research progress on the structure, working principles, pre-treatment technology, and recycling and regeneration technology of positive electrode materials in lithium-ion batteries. It provides a detailed overview of recent research on lithium battery recycling and puts forward suggestions for the future lithium battery recycling industry.

## 2. Structure and Working Principle of LIBs

### 2.1. Structure of LIBs

**Figure 2:**
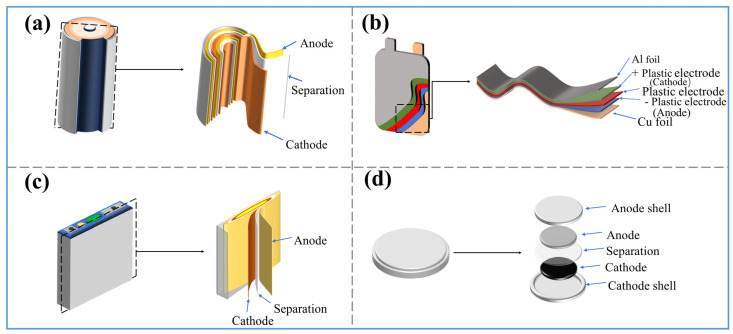
Common structures of lithium-ion batteries: (**a**) cylindrical shape; (**b**) sheet shape; (**c**) square shape; (**d**) button shape.

The lithium battery primarily consists of positive electrode material, negative electrode material, electrolyte, separator, and battery casing [24]. The positive electrode active materials mainly include lithium cobalt oxide (LiCoO_2_), lithium nickel manganese cobalt oxide (LiNiMnCoO_2_, NMC), lithium iron phosphate (LiFePO_4_), lithium manganese oxide (LiMn_2_O_4_), and lithium nickel cobalt aluminum oxide battery (NCA), etc. The composition and content of each component in lithium batteries are shown in Table 2. The lithium battery anode is mainly composed of copper foil or aluminum foil coated with active material through a binder. Copper foil or aluminum foil acts as a current collector, transferring the current from the electrode to the external circuit of the battery. The binder’s function is to firmly bond the particles together to form a conductive electrode structure. Common binders include polyvinylidene fluoride (PVDF) [25], polytetrafluoroethylene (PTFE) [26], and carboxymethyl cellulose (CMC) [27]. Currently, the main anode active material is graphite. The electrolyte of lithium-ion batteries mainly consists of organic carbonate solvents, such as ethylene carbonate (EC), propylene carbonate (PC), dimethyl carbonate (DMC), methyl acrylate carbonate (MAPC), and 1,2-dimethoxyethane (DME), etc. [28,29,30]. These organic carbonate solvents are usually mixed with lithium salts (such as LiPF_6_, LiBF_4_, etc.) to form the electrolyte [31]. The separator of LIBs is a critical component between the cathode and anode, mainly preventing direct electron flow while allowing lithium ions to pass through during charge and discharge. Its main materials are polyethylene (PE), polypropylene (PP), and other polymers [32,33]. The casing of lithium-ion batteries, which protects the internal structure of the battery from external environmental influences, is usually made of metal or plastic. Figure 2 shows the structure of common lithium-ion batteries (pouch, cylindrical, and prismatic).

### 2.2. Working Principle of LIBs

The basic structure of a lithium battery mainly includes the anode, cathode, and electrolyte. Lithium ions travel back and forth between the cathode and anode through the electrolyte. The electrolyte generally does not participate in the chemical reactions within the lithium battery, but its type significantly affects the performance of the lithium battery [40]. Liquid electrolytes and solid-state electrolytes each have unique properties that influence battery characteristics such as energy density, charge/discharge rates, cycle life, and safety [41,42]. Since there is relatively little research on the recycling of solid-state electrolyte batteries at present, this will not be discussed in this review. The anode and cathode of the battery need to be separated by a separator, whose microporous structure allows lithium ions to pass through while preventing the flow of electrons, thus preventing direct electron flow between the two electrodes from causing a short circuit [43].

The cathode of a lithium battery typically consists of layered compounds such as lithium cobalt oxide (LiCoO_2_) or lithium nickel manganese cobalt oxide (LiNiMnCoO_2_). These layered cathode structures undergo intercalation and deintercalation reactions of lithium ions during the charging and discharging processes [44].

**Figure 3:**
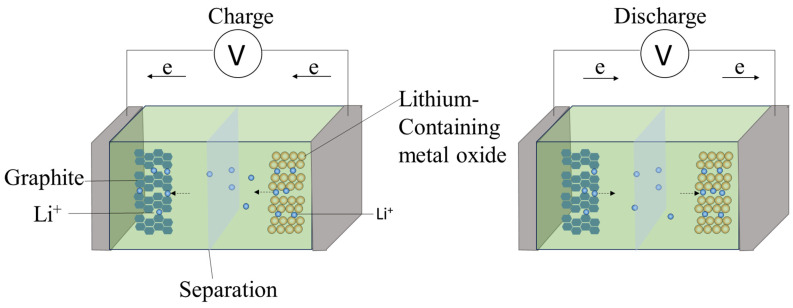
Working principle of LIBs.

As illustrated in Figure 3, during the charging process, lithium ions exit the cathode material, pass through the electrolyte, and embed themselves in the anode material. At the same time, electrons flow from the cathode to the anode in the external circuit, opposite to the direction of ion flow, to maintain the overall electrical neutrality of the battery. The discharging process is the reverse: lithium ions exit the anode, travel through the electrolyte to the cathode material, and embed themselves in its lattice structure, while electrons flow from the anode to the cathode through the external circuit.

Taking the common lithium metal oxides-polymer separator-graphite system battery as an example, the chemical reactions occurring during the operation of a lithium battery are described as follows:

(i) Charging Process

The reaction at the cathode occurs as shown in Equation (1), causing lithium ions to move out of lithium-containing metal oxide. 


(1) $$LiMO_{2}\to Li_{1-x}MO_{2}+xLi^{+}+xe^{-}$$

The reaction at the anode, as shown in Equation (2), involves the insertion of lithium ions into graphite (the layered structure in graphite is called lithiated graphite, LiC_6_).


(2) $$6C+xLi^{+}+xe^{-}\to Li_{x}C_{6}$$

(ii) Discharging Process

The reaction at the cathode, as shown in Equation (3), involves lithium ions being inserted back into the lithium-containing metal oxide.


(3) $$xLi^{+}+Li_{1-x}MO_{2}+xe^{-}\to LiMO_{2}$$

The reaction at the anode, as shown in Equation (4), entails lithium ions moving out of the graphite.


(4) $$Li_{x}C_{6}\to xLi^{+}+6C+xe^{-}$$

The total reaction of charging and discharging is shown in Equations (5) and (6).


(5) $$LiMO_{2}+6C⇌Li_{1-x}MO_{2}+Li_{x}C_{6}$$

(6) $$\left(LiFePO_{4}⇌xFePO_{4}+(1-x)LiFePO_{4}+xLi^{+}+xe^{-}\right)$$

In the equations, M represents Ni, Co, Mn or other metal composites.

## 3. Disassembly and Pretreatment of Spent LIBs

In general, lithium-ion batteries become ineffective after approximately 5–8 years of use [24], and spent LIBs should be recycled. On one hand, spent LIBs contain a large amount of heavy metals such as nickel, cobalt, manganese, etc. Improper disposal of these metals can lead to serious pollution of soil, plants, and the ecological environment [45]. Investigations show that cobalt, a heavy metal, poses a significant health hazard and is carcinogenic. Its strong permeability allows it to penetrate the skin, leading to lung and gastrointestinal diseases [46]. In addition to heavy metals, the electrolytes in spent LIBs contain chemicals like lithium salts, organic solvents, and additives, which harm the environment and human health. The main electrolyte component, LiPF_6_, decomposes in the presence of water to produce harmful substances such as POF_3_ and HF, and releases toxic P_2_O_5_ during combustion, causing severe pollution [47]. The organic solvents in LIBs are volatile and hard to degrade, potentially polluting the atmosphere, soil, and water. Global demand for lithium is also rising, from 12,000 tons in 2020 to a projected 25,000 tons by 2025 [48]. Lithium resources are becoming scarce due to high extraction costs and uneven geographic distribution. Traditional methods of extracting lithium from ores and brine are unlikely to meet the growing demand. Therefore, recycling spent lithium-ion batteries (LIBs) has become essential for obtaining lithium resources, reducing environmental pollution, and addressing resource scarcity.

**Figure 4:**
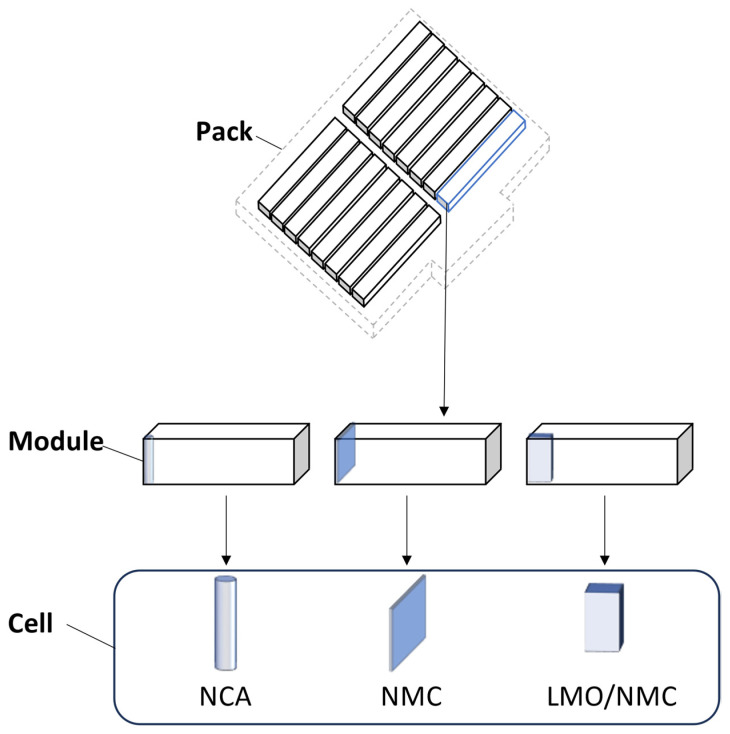
The form of cells in battery packs.

Recycling spent lithium-ion batteries (LIBs) not only reduces pollution but also increases metal resources, alleviating resource scarcity. The pretreatment process is of great significance to the treatment of solid waste [49]. Before valuable elements from spent LIBs can be recycled, they undergo operations such as classification, discharge, disassembly, and material separation [50]. Current research focuses on recycling and resource utilization starting from the cell stage. As shown in Figure 4, taking automotive power lithium-ion batteries as an example, the entire battery pack consists of modules composed of battery cells. The primary task of recycling spent battery cells is to disassemble the cells from the pack. However, for effective resource utilization and cost-effectiveness, it is necessary to wisely choose which batteries to disassemble. Identifying and screening cells that have lost functionality is crucial, which requires electrochemical performance testing to determine which cells need to be disassembled for recycling. At the same time, cells or modules with good electrochemical performance can be directly reused (such as by reassembling them into new battery packs) without disassembly. This approach saves costs, maximizes battery lifespan, and reduces environmental impact.

**Figure 5:**
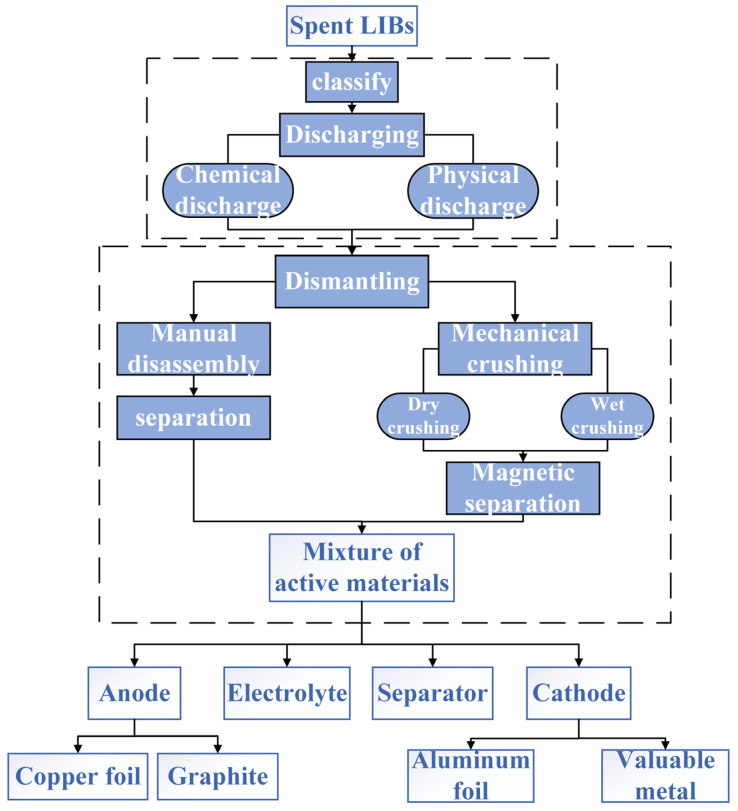
Conventional preprocessing process of spent LIBs.

Currently, there is no unified standard for the preprocessing process of recycling or reusing spent LIBs, and the process is complex. The general process flow is shown in Figure 5. After lithium-ion batteries are classified, they need to undergo chemical or physical discharge. After discharge, manual or mechanical disassembly can be conducted, followed by various methods of separation to obtain positive electrode active materials.

### 3.1. Classify

In the recycling of spent LIBs, the classification and analysis of the overall battery condition are extremely important. Many recycling companies classify batteries based on their chemical composition or positive electrode material type (LFP, NCM, etc.) to optimize the performance of wet metallurgical processes [51]. This approach typically starts with understanding the working principle of the battery and then determining the electrochemical parameters required for battery chemical reactions. However, obtaining the internal electrochemical parameters of the battery is often challenging as it may damage the battery. Therefore, estimating the chemical properties of the battery often relies on characteristics such as size, weight, and voltage. Most batteries do not provide written information to identify their chemical composition and internal structure, or aging may blur this information, further increasing the difficulty of battery classification. In recent years, researchers have used cutting-edge methods such as machine learning [52] or deep learning [53] to construct electrochemical models to determine the electrochemical parameters of batteries.

### 3.2. Discharge

Before dismantling and crushing, spent LIBs usually still have some charge, which may pose a certain risk to the dismantling and crushing process [54]. When dismantling and crushing charged spent LIBs, it is easy for contact to occur between the positive and negative electrodes, leading to a short circuit. The short-circuit current will cause a sharp increase in the internal temperature of the battery, potentially causing the synthesis and release of heat, toxic, and corrosive compounds and even leading to combustion or explosion. At the same time, the electrolyte and flammable solvent inside the battery may also be ignited by the short-circuit current, causing the battery to explode [55]. Therefore, before dismantling the battery case, spent LIBs must be discharged to reduce the risk of spontaneous combustion or short circuits.

The discharge principle of lithium-ion batteries is to consume the battery power through the resistance connected between the positive and negative electrodes. Currently, there are mainly two feasible methods: physical discharge [56] and chemical discharge [57]. The use of conductive powder particles for discharge is a common method of physical discharge, usually achieved using graphite and metal chips or powders. By using a resistor to short-circuit the positive and negative electrodes, the internal power of the battery can be quickly consumed, and this technology is widely used in small-capacity batteries because it provides a fast discharge rate. The short-circuit operation allows energy to be recovered from larger-sized batteries, but cooling equipment needs to be installed because a large amount of heat is generated during rapid discharge. In addition, due to the increase in internal pressure of the battery, electrolyte leakage and local overheating may occur, so careful handling is required. Chemical discharge is the mainstream method of discharging spent LIBs currently. In this process, a conductive solution is generally used to discharge the lithium battery, and this solution is usually composed of salt, acid, or alkali. Immersing the positive and negative electrodes of the battery in the conductive solution will cause them to short-circuit. This technology is generally considered safe because the solution can effectively absorb energy. However, various chemical reactions will occur during the discharge process, and if the battery is damaged, the solution may be contaminated. Sodium chloride is a common conductive solution for battery discharge, and research has shown that immersing spent LIBs in a NaCl solution for 45 h can complete the discharge, and a 10 wt% NaCl solution can complete the discharge of spent LIBs within 18 h [58]. When using organic solvents, acid solutions, and alkaline solutions for discharge, attention should be paid to avoiding damage to the lithium battery to ensure that discharge performance is not affected.

### 3.3. Dismantling

In the processing of spent LIBs, dismantling is a key step, and currently, there are two main methods: manual dismantling [59] and robot dismantling [60]. Manual dismantling relies on professional technicians who use various tools and equipment, such as pliers, wrenches, and safety gloves, to gradually disassemble the batteries. This method offers high flexibility and strong controllability, allowing for careful separation of battery components. However, it also has significant disadvantages, including high labor intensity and potential safety hazards [61,62]. On the other hand, robotic dismantling uses automated equipment and robotic technology to disassemble and separate batteries through program-controlled tools, such as mechanical arms. This method boasts high efficiency, speed, and enhanced safety. However, robots cannot handle the different designs and layouts of lithium-ion batteries with a very high success rate [63,64].

### 3.4. Crushing

The process of battery fragmentation is the mechanical disassembly of battery packs to obtain battery materials containing valuable metals. Different crushing procedures produce fragments of different sizes and shapes, which have a significant impact on the subsequent separation process. Compared to manual dismantling, the use of mechanical crushing and other technologies can improve processing efficiency and capacity. Crushing is usually carried out in stages, and multiple stages of crushing are performed to ensure uniform size distribution and content distribution of the generated battery fragments. Multistage crushing helps improve the effectiveness of subsequent separation processes [65].

In industrial processes, dry crushing is commonly used for fragmenting batteries. This method sorts batteries before batch crushing to maintain particle consistency and improve separation efficiency. It leverages the selective crushing characteristics of spent LIBs, preventing excessive fragmentation of other components, thereby enhancing the purity and dispersion of electrode materials and facilitating subsequent purification and regeneration [66]. However, this process is time-consuming, labor-intensive, and requires expensive equipment, along with a dust control system to prevent pollution. Initially, batteries are preliminarily crushed to expose and separate components, followed by magnetic separation to extract iron particles and further crushing to achieve the desired particle size. Mechanical crushing in inert and low-temperature environments has gained attention due to the risk of short-circuiting and reactions with air during the process. Inert environments can suppress such reactions, while low temperatures make the plastic casing brittle, simplifying crushing. This method reduces fire risk, making it a preferred choice in recycling plants for handling large quantities of batteries safely. Customizable inert environments, using gases like argon, nitrogen, and carbon dioxide, prevent oxidation-reduction reactions and avoid spontaneous combustion or hazardous gas production [67,68].

Wet crushing involves crushing lithium-ion batteries in water or other solutions to prevent reactions with electrolytes [58]. This method reduces dust generation and prevents equipment clogging, while also acting as a cleaning agent and flame retardant by absorbing heat and reducing combustion and explosion risks associated with dry crushing. Water’s role in grinding is crucial, as dry grinding leads to greater surface roughness, particle agglomeration, and surface oxidation, impacting subsequent separation processes [69]. High-speed, high-pressure water can be used for cutting batteries, producing less noise, consuming less energy, and shortening the processing cycle. However, harmful compounds such as binders and electrolytes are discharged into the water, complicating wastewater treatment and increasing operating costs.

After being crushed, spent LIBs exist in the form of mixed materials. At this stage, the crushed mixture can be directly smelted through pyrometallurgy to obtain elemental metals and their oxides. However, when conducting more detailed recovery research on the positive electrode materials, component separation of the crushed mixture is generally required.

### 3.5. Separation

After deactivation, spent LIBs require component separation to recover enriched active cathode powder. These batteries can be manually disassembled into plastic/metal casings, polymers, anode foils, and cathode foils. Modern recycling facilities prefer using crushing processes followed by magnetic separation to isolate metal parts, enhancing processing capacity. Fine crushing and screening further recover active cathode materials, resulting in higher-grade battery materials. Common methods for separating the cathode active material from the aluminum foil and removing the organic binder include mechanical separation [70], solvent dissolution [71,72,73], and high-temperature separation [55]. 

After a series of pretreatment processes such as discharging, dismantling, and separation of spent LIBs, if valuable metals in the batteries are desired, different recycling processes can be used to extract valuable metals. NCM, LCO, LMO, and NCA lithium-ion batteries typically contain metals such as nickel, cobalt, manganese, and aluminum, which are present in relatively high concentrations in the batteries. Because these metals have high commercial value and are important in the circular economy, recovering these metals from such batteries is crucial for efficient resource utilization. Previously, researchers like Fan [74] and Xuan et al. [75] simplified the chemical reactions of recycling spent NCM ternary cathode materials as LiMO_2_ (where M represents Ni, Co, Mn). In contrast, the chemical composition of LFP batteries is quite different from the former four, with the main component being LiFeO_4_, and not containing higher-value metals such as nickel and cobalt. Therefore, based on the chemical composition of battery cathode materials and metal components, this paper classifies NCM, LCO, LMO, and NCA as one category and LFP as another. In the subsequent sections, this paper will introduce chapters on pyrometallurgical recovery, hydrometallurgical recovery, and cathode material regeneration in detail, comparing the differences between nickel–cobalt–manganese batteries (NCM, LCO, LMO, NCA) and LFP batteries in applying these methods and comparing the advantages and disadvantages of different process routes.

## 4. Pyrometallurgical Process

This review paper used pyrometallurgical processes for spent LIBs in recent years. Then, it provides a detailed discussion of the pyrometallurgical processes available for nickel–cobalt–manganese batteries and LFP batteries. Pyrometallurgy is a common method for recycling spent LIBs. It involves high-temperature treatment and thermal decomposition to burn off the organic materials and polymers in the batteries, thereby extracting valuable metal elements. Pyrometallurgical recycling has the advantages of high processing efficiency and metal recovery rates. It is suitable for large-scale production and processing of various types of spent LIBs. The two main methods are high-temperature smelting and reduction roasting.

### 4.1. High-Temperature Smelting

The core of high-temperature smelting is to remove the organic materials from spent LIBs through high temperatures. The internal metal components, such as the anode and cathode, are heated to high temperatures and melted. During this process, the different melting points of the metals cause them to dissolve and mix in the smelting furnace, being recovered in the form of Ni, Co, Mn, and Cu alloys [76]. High-temperature smelting involves two important processes: (1) Thermal treatment of the materials at lower temperatures to promote the evaporation of the electrolyte and prevent overpressure explosions caused by intense heating. This step aims to avoid the sudden evaporation of the electrolyte, which can generate abnormal gas pressure, potentially damaging the internal structure of the battery and leading to explosions. (2) Further heating of the materials at higher temperatures to achieve a molten state for subsequent processing. This method can recover various types of spent LIBs with different chemical compositions without requiring extensive pretreatment. Both modules and cells can be directly fed into the furnace for reaction [19]. A well-designed slag system plays a crucial role in the high-temperature smelting method. An effectively designed slag system can efficiently separate the metallic and non-metallic parts, enhancing the efficiency of metal recovery. This maximizes the utilization of resources from spent LIBs and reduces the generation of waste [77]. In this method, the carbon and aluminum in spent LIBs act as reducing agents. The reactions can be summarized by Equation (7):


(7) $$LiMO_{2}+C+Al\to Li_{2}O+M+CO+Al_{2}O_{3}$$

In Equation (7), M represents Ni and Mn. During this process, the aluminum in spent lithium batteries forms slag as Al_2_O_3_ [78]. In high-temperature smelting, common slag systems include FeO-SiO_2_-Al_2_O_3_ and MnO-SiO_2_-Al_2_O_3_ [79,80]. Judging from the current studies, after the smelting process of spent LIBs, transition metals preferentially concentrate to form a molten alloy phase, which settles to the bottom of the furnace and enters the molten metal pool. Subsequently, valuable metals are further recovered from the alloy using hydrogen metallurgy techniques [55]. Lithium typically exists in the form of dust in flue gas ducts. To extract lithium ions, water leaching is usually required, followed by converting them into lithium carbonate or lithium phosphate precipitates [81].

### 4.2. Reduction Roasting

Reduction roasting refers to the process where the active materials of spent LIBs’ cathodes are reduced to metals or other relevant substances in a high-temperature reducing atmosphere [82]. Reduction roasting can be further divided into carbon thermal reduction (CTR) and salt-assisted roasting. Using CTR to treat recovered cathode active materials from lithium batteries entails heating the active cathode materials alongside a reducing agent (such as graphite inherent in lithium battery materials or additional added charcoal). This process generates a mixture of carbon residues and alloys/intermediate compounds (containing impure metals/oxides), providing raw materials for further refinement [83]. The reactions of CTR can be summarized approximately by Equations (8) and (9).


(8) $$LiMO_{2}+C\to Li_{2}CO_{3}+M$$

(9) $$LiMnO_{2}+C\to Li_{2}CO_{3}+MnO$$

In Equation (7), M represents Ni and Co. The residue obtained after CTR can generally be subjected to water leaching, followed by wet magnetic separation or mechanical separation, to isolate Li_2_CO_3_, metallic elements, and residual carbon [84]. When oxides are present in the residue, lithium products can be recovered first. Then, the residue can undergo roasting to remove carbon and obtain the oxides [85].

Salt-assisted roasting is an emerging method developed after carbon thermal reduction. In traditional carbon thermal reduction, the Li_2_CO_3_ produced is insoluble in water, resulting in a lower lithium recovery rate. Salt-assisted roasting can transform various metal elements into water-soluble products, thereby improving the recovery efficiency. Depending on the reagents used, salt-assisted roasting can be categorized as sulfated roasting [86], nitration roasting [87], chlorination roasting [88,89], etc. The products of sulfated roasting typically include metal oxides, sulfates, or sulfides. In most cases, the added sulfiding agent undergoes redox reactions at high temperatures, and the decomposed SO_2_ (some sulfiding agents can decompose into SO_2_ and O_2_) reacts with the cathode powder along with O_2_. The product after sulfate roasting typically requires water leaching to remove impurities. Subsequently, by adjusting the pH, oxides of Ni, Co, and Mn can be obtained. Finally, adding Na_2_CO_3_ yields Li_2_CO_3_. For LiFePO_4_, it only needs filtration to separate LiSO_4_ and FePO_4_, then adding Na_2_CO_3_ to obtain Li_2_CO_3_ [90,91,92]. The products of nitration roasting typically include metal oxides or nitrates. The mechanism usually involves the high-temperature decomposition of the nitriding agent, producing NO, which, along with O_2_, reacts with the cathode material to undergo redox reactions. Currently, there is limited research on how to recover products after nitric acid roasting. According to the study [93], lithium can be carbonized with Na_2_CO_3_ to form Li_2_CO_3_, while the remaining metal solution requires acid leaching followed by extraction or electrodeposition to obtain metal products. During nitration roasting, nitric oxide gas generated in the process can be converted into nitric acid through oxidation agents, catalysts, or pressurized adsorption [93]. The products of chlorination roasting typically include chlorides. The generated chlorine gas can be neutralized in a sodium hydroxide solution or recycled back into the reactor [55]. The residue after roasting typically consists of various metal chlorides, each requiring different temperatures for chlorination. By using different temperature gradients, specific metal solutions can be obtained, which can then be processed through solvent extraction or other methods to recover the desired metal products [88]. The core process involves the high-temperature decomposition of the chlorinating agent, producing Cl_2_, which, along with O_2_, interacts with the cathode material. Because salt-assisted roasting involves a variety of auxiliary reagents and varies in its effects on nickel–cobalt–manganese batteries and LFP cathode materials, this paper does not summarize their reactions here. Detailed information can be found in the following sections.

### 4.3. Pyrometallurgy of Nickel–Cobalt–Manganese Cathodes 

**Figure 6:**
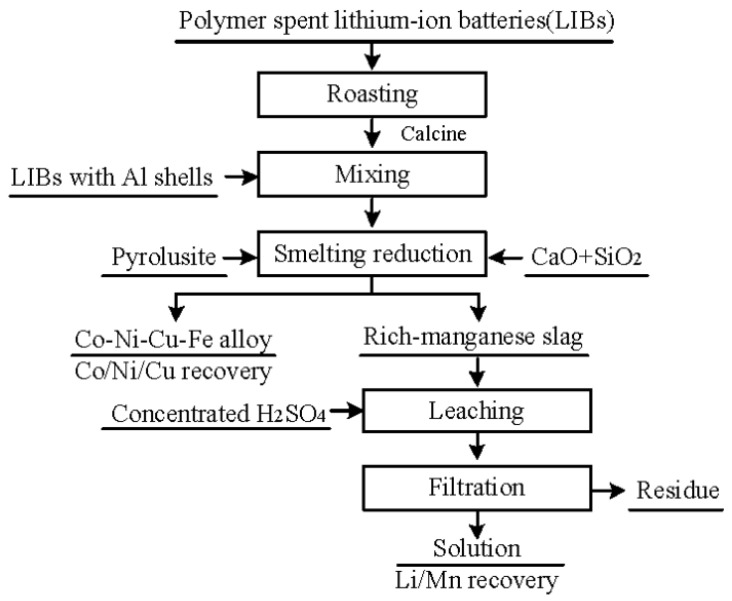
Flowchart of recovering Co, Ni, Cu, Mn, and Li from spent LIBs by smelting [79], Elsevier.

**Figure 7:**
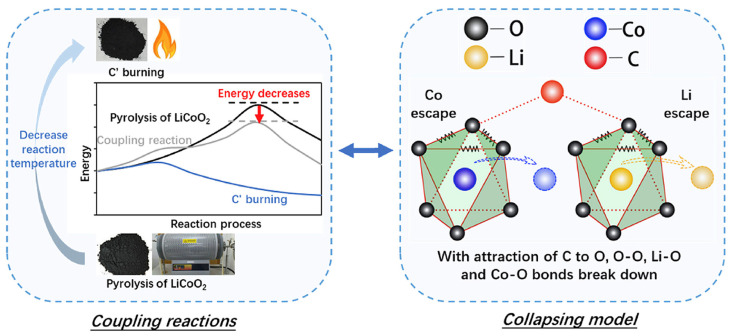
Coupling reaction mechanism and collapse model in carbothermal reduction roasting [97], Elsevier.

High-temperature smelting is commonly used for recycling this type of battery and is widely applied on an industrial scale due to its high productivity and simple operation [94]. In industrial smelting, the CaO-SiO_2_-Al_2_O_3_ slag system is common. However, its high viscosity can affect the mixing and transfer of waste and metals, leading to operational difficulties or uneven chemical reactions. It may also have strong oxidizing properties, which can cause losses or interfere with the smelting process for some easily oxidizable elements or compounds. Adding appropriate amounts of cobaltite can improve the recovery efficiency of this process [95,96]. However, due to the scarcity of cobaltite resources, the application of this process is limited. To address this issue, Ren et al. [79] proposed a novel slag system FeO-SiO_2_-Al_2_O_3_ for recovering NCA, using waste copper slag as the sole flux. Copper slag resources are more abundant than cobaltite, mainly composed of iron ore, with a total FeO and SiO_2_ content of over 70%. Therefore, copper slag can provide both FeO and SiO_2_ for the smelting process as a slagging agent. The results show that under optimal conditions with a slag agent to battery mass ratio of 4.0:1, a smelting temperature of 1723 K, and a smelting time of 30 min, the recovery rates of cobalt, nickel, and copper were 98.83%, 98.39%, and 93.57%, respectively. It is noteworthy that although the FeO-SiO_2_-Al_2_O_3_ slag system is more economical than CaO-SiO_2_-Al_2_O_3_, both systems cannot recover Li and Mn, as these elements enter the insoluble slag during smelting. To solve this problem, they conducted further research using a MnO-SiO_2_-Al_2_O_3_ slag system, producing a Co-Ni-Cu-Fe alloy and lithium-rich manganese slag. Subsequent leaching of the slag resulted in Li and Mn recovery rates of 94.85% and 79.86%, respectively. The detailed recovery process is shown in Figure 6 [80]:

Another pyrometallurgical approach for recovering valuable metals from spent nickel–cobalt–manganese batteries is reduction roasting. For these batteries, common reduction roasting methods include carbon thermal reduction (CTR) and salt-assisted roasting. The coupling reaction mechanism and collapse model of carbon thermal reduction are shown in Figure 7:

Li et al. [84] subjected a blend of LCO and graphite to roasting at 1000 °C in an oxygen-free atmosphere for 30 min. The roasting products were a mixture of Co, Li_2_CO_3_, and graphite. The reactions that occurred are shown in Equations (10)–(12):


(10) $$4LiCoO_{2}+2C\to 4Co+2Li_{2}CO_{3}+O_{2}(g)$$

(11) $$2LiCoO_{2}+2C\to 2Co+Li_{2}CO_{3}+CO(g)$$

(12) $$4LiCoO_{2}+3C\to 4Co+2Li_{2}CO_{3}+CO_{2}(g)$$

Following wet magnetic separation, cobalt, lithium, and graphite achieved recovery rates of 95.72%, 98.93%, and 91.05%, respectively. In subsequent studies, they demonstrated the feasibility of this roasting method for LMO and NCM materials. The LMO materials were roasted at 800 °C for 45 min, during which the LMO active materials underwent reduction by graphite in the mixture, resulting in the formation of Li_2_CO_3_ and MnO. Through subsequent water leaching and mechanical separation processes, 99.13% of lithium was recovered, while the filter residue was subjected to calcination to eliminate carbon content, resulting in Mn_3_O_4_ with a purity of 95.11%. However, for NCM cathode materials, the recovery rate of Li using this method was only 66.25% [98]. Their research indicates that carbon thermal reduction is most suitable for processing LCO cathode materials, as this method efficiently extracts high-value metallic Co and recovers Li and graphite at high rates. Although carbon thermal reduction can efficiently extract valuable metals from spent lithium batteries, it produces pollutant gases CO and greenhouse gas CO_2_. To address this issue, Li et al. [99] used CTR and hydrogen thermal reduction (HTR) to process NCA materials. The results showed that although HTR’s metal extraction efficiency is lower than CTR’s, it does not produce CO_x_ gases. They believe that although the material cost of hydrogen reduction is higher than that of carbon reduction, hydrogen reduction has greater potential for industrial application from a comprehensive analysis perspective. We argue that hydrogen poses a risk of explosion, leading to safety accidents and that strict management of hydrogen is necessary for industrial application. Currently, research on HTR is limited, and more studies are needed to prove the feasibility of this method.

As mentioned earlier, compared to CTR, salt-assisted roasting improves recovery efficiency and reduces the emission of toxic gases by generating water-soluble salts. Chlorination roasting, sulfation roasting, and nitration roasting in salt-assisted roasting can all be used for pyrometallurgical recovery of nickel–cobalt–manganese cathode materials. When chlorination roasting nickel–cobalt–manganese cathodes, the products are usually soluble metal oxides. Common chlorinating agents include NaCl, HCl, and Cl_2_. Xiao et al. [100] roasted NH_4_Cl with LMO cathode powder and the reaction that occurred is shown in Equation (13):

(13) $$\begin{array}{l} 2LiMn_{2}O_{4}(s)+10NH_{4}Cl(g)\to 4MnCl_{2}(s)+2LiCl(s)+8H_{2}O(g)+N_{2}(g)\\ +8NH_{3}(g) \end{array}$$

Transition metal elements are recovered as metal oxides through filtration, and Li is recycled as Li_2_CO_3_ by adding Na_2_CO_3_, achieving a Li recovery rate of 90.04%. Compared to other chlorinating agents, NH_4_Cl has a lower efficiency for lithium extraction and produces NH_3_ exhaust gas that requires additional treatment. Therefore, it cannot be considered the best chlorinating agent for NCM cathode materials. Wei et al. [101] used CaCl_2_ as a chlorinating agent in the roasting process of LCO. The reactions that occurred are shown in Equations (14) and (15):

(14) $$LiCoO_{2}=1/2LiCl+1/3Co_{3}O_{4}+1/12O_{2}(g)$$

(15) $$LiCoO_{2}+CaCl_{2}\cdot 6H_{2}O=LiCl+1/3Co_{3}O_{4}+3H_{2}O+1/2CaO+1/12O_{2}(g)$$

In an air atmosphere, raising the temperature causes CaCl_2_ to decompose into Cl_2_ and HCl. 

During the process, the layered structure of LCO disintegrates, yielding Co_3_O_4_ and Li_2_O. At 750 °C under mild chlorination conditions, lithium ions are liberated and react with Cl^−^ to produce water-soluble LiCl, while the Co-O octahedral structure remains relatively intact, resulting in water-insoluble Co_3_O_4_. The outcomes reveal that 98.12% of Co is obtained as Co_3_O_4_, and 99.49% of Li is selectively obtained as LiCl. Through the addition of Na_2_CO_3_, LiCl can be further recovered as battery-grade Li_2_CO_3_. It is important to note that during the roasting process, the amount of chlorinating agent can be adjusted to control the chlorine partial pressure in the reaction system. This ensures that the chlorination reaction occurs under low chlorination conditions, preventing the release of excess Cl_2_ and avoiding environmental pollution.

The mechanism of sulfation roasting involves using sulfating agents such as H_2_SO_4_, (NH_4_)_2_SO_4_, Na_2_SO_4_, or NaHSO_4_·H_2_O to convert the Li in cathode materials into water-soluble Li_2_SO_4_. Similar to NH_4_Cl, using (NH_4_)_2_SO_4_ in a single-step roasting of cathode materials results in an unsatisfactory Li recovery rate [102]. To address this issue, Wei et al. [103] proposed a two-step roasting method using (NH_4_)_2_SO_4_. Initially, the LCO structure undergoes decomposition, leading to the conversion of a portion of Li and Co into Li_2_SO_4_ and CoSO_4_, with sulfur stored and retrieved as SO_4_^2−^. Subsequently, CoSO_4_ serves as a catalyst, reacting with the remaining LCO as temperature rises to generate water-soluble Li_2_SO_4_, while CoSO_4_ transforms into water-insoluble Co_3_O_4_. Following this, lithium is preferentially extracted through subsequent water leaching, resulting in 98.75% extraction efficiency and the production of battery-grade Li_2_CO_3_. Additionally, 99.32% of Co is recovered as Co_3_O_4_. However, similar to NH_4_Cl, the use of (NH_4_)_2_SO_4_ also results in the unavoidable production of NH_3_. Among sulfating agents, H2SO4 can maintain a high recovery rate and selectivity for Li, while being cleaner than other sulfating agents. Lin et al. [92] pretreated LCO with H_2_SO_4_ at 393 K, primarily to convert Co in the LCO to CoSO_4_. In the subsequent thermal treatment step, similar to the study by Wei et al. [103], CoSO_4_ continues to selectively react with the remaining LiCoO_2_ until it is fully depleted. The overall reaction of this experimental process is shown in Equation (16):

(16) $$\begin{array}{l} 12LiCoO_{2}(s)+6H_{2}SO_{4}(l)\to 6Li_{2}SO_{4}(s)+4Co_{3}O_{4}(ll,lll,s)+\\ 6H_{2}O(g)+O_{2}(g) \end{array}$$

Testing showed that 99.3% of lithium and 98.7% of cobalt were reclaimed as Li_2_CO_3_ and Co_3_O_4_, with purities reaching 99.89% and 99.95%, respectively. The advantage of using H_2_SO_4_ as a sulfating agent is that by controlling the amount of sulfuric acid, sulfur can be recycled in the form of SO_4_^2−^, avoiding SO_x_ emissions that pollute the environment. However, its corrosiveness may increase industrial costs. (The reference is the same as [92]).

The core of nitration roasting is to use nitrating agents to extract Li from waste batteries in the form of LiNO_3_. Based on the current research on roasting waste lithium batteries, using carbothermic reduction roasting, chlorination roasting, and sulfation roasting requires maintaining roasting temperatures above 600 °C to ensure the effective separation of lithium and other valuable metals. Nitration roasting can extract Li at relatively lower temperatures because the relevant nitrates of metals other than Li in waste lithium batteries decompose into insoluble oxides at 125–250 °C [104,105], while LiNO_3_ decomposes at around 600 °C [106]. Based on this background, Peng et al. [93] used HNO_3_ as a nitrating agent to roast waste NCM cathode powder at 250 °C. Due to the complexity of the components in NCM cathode materials, the Ni, Co, and Mn components are assumed to be in the forms of LiNiO2, LiCoO_2_, and LiMnO_2_. The reaction equations are shown as Equations (17)–(22):

(17) $$LiCoO_{2}+4HNO_{3}\to LiNO_{3}+Co{\left(NO_{3}\right)}_{2}+NO_{\left(g\right)}+O_{2(g)}+H_{2}O_{\left(g\right)}$$

(18) $$LiNiO_{2}+4HNO_{3}\to LiNO_{3}+Ni{\left(NO_{3}\right)}_{2}+NO_{\left(g\right)}+O_{2(g)}+H_{2}O_{\left(g\right)}$$

(19) $$LiMnO_{2}+4HNO_{3}\to LiNO_{3}+Mn{\left(NO_{3}\right)}_{2}+NO_{\left(g\right)}+O_{2(g)}+2H_{2}O_{\left(g\right)}$$

(20) $$Fe+4HNO_{3}\to Fe{\left(NO_{3}\right)}_{3}+NO_{\left(g\right)}+2H_{2}O_{\left(g\right)}$$

(21) $$Al+4HNO_{3}\to Al{\left(NO_{3}\right)}_{3}+NO_{\left(g\right)}+2H_{2}O$$

(22) $$1.5Cu+4HNO_{3}\to 1.5Cu{\left(NO_{3}\right)}_{2}+NO_{\left(g\right)}+2H_{2}O$$

**Figure 8:**
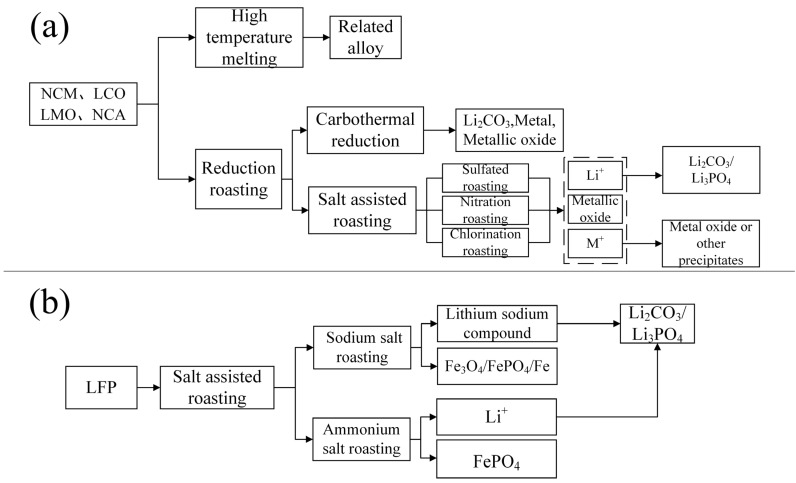
(**a**) Pyrometallurgy process of nickel–cobalt–manganese cathode materials; (**b**) Pyrometallurgy process of LFP cathode materials.

After filtration, the filtrate reacts with Na_2_CO_3_ solution to produce battery-grade Li_2_CO_3_. The lithium recovery rate is as high as 93%. The Co, Ni, and Cu residues require acid leaching for recovery. Since Peng et al. [93] did not provide detailed descriptions of Co, Ni, and Cu, and this chapter mainly focuses on pyrometallurgical recovery processes, this aspect will not be elaborated upon here. The nitration roasting process often produces NO_x_ gases, but these can be recovered as nitric acid through pressurized acid adsorption, catalysts, or oxidants (such as H_2_O_2_ and ozone), forming a closed-loop process for recycling spent LIBs through nitration roasting. The general pyrometallurgical process for nickel–cobalt–manganese cathode materials can be referred to in Figure 8a.

### 4.4. Pyrometallurgy of LFP Cathode

As mentioned earlier, when using high-temperature smelting to recycle spent LIBs, lithium is lost due to the formation of insoluble slag. Since lithium is the most valuable metal in LFP cathode materials, using high-temperature smelting to recover LFP is not an economical approach. Furthermore, owing to the thermodynamically stable olivine structure of LFP, conventional nitration/chlorination processes face challenges in carbon reduction to iron or conversion into iron oxides and salts. In recent studies, compounds related to sodium are commonly used for auxiliary roasting of spent LFP. Roasting with sodium bisulfate is an effective method to separate lithium from the LiFePO_4_ structure. Zhang et al. [107] introduced NaHSO_4_·H_2_O into spent LFP powder for mixed roasting with oxygen gas, and the optimal roasting condition was found to be 600 °C for 1 h. The overall reaction is depicted by Equation (23):

(23) $$\begin{array}{c} LiFePO_{4}+NaHSO_{4}\cdot H_{2}O+C+5/4O_{2}(g)\\ =1/3Fe_{2}O_{3}+1/3FePO_{4}+LiNaSO_{4}+CO_{2}(g)+3/2H_{2}O(g)+1/3P_{2}O_{5} \end{array}$$

After water immersion, the leaching rate of Li achieves an impressive 98.12%, whereas the leaching rates of Fe, Al, and P are all less than 1%. Although this process achieves excellent lithium extraction results, the generated P_2_O_5_ exists in a gaseous form. P_2_O_5_ possesses strong corrosiveness and toxicity, which can cause harm to human health if not handled properly. One of the key aspects in the pyrometallurgical recovery of LFP is the need to disrupt its olivine structure as much as possible. Based on this, Zhang et al. [108] utilized Na_2_CO_3_ or NaOH as fracture agents for Fe-PO_4_, aiding in the carbon thermal reduction of LFP. During the calcination process, LFP initially reacts with Na_2_CO_3_ or NaOH to form iron oxides, which are then reduced to iron, separable from the calcination products through magnetic separation. The reactions occurring are shown in Equations (24) and (25), respectively:

(24) $$\begin{array}{l} 3LiFePO_{4}+3Na_{2}CO_{3}+1.5C=3Fe+NaLi_{2}PO_{4}+LiNa_{5}{\left(PO_{4}\right)}_{2}+\\ 4.5CO_{2}(g) \end{array}$$

(25) $$\begin{array}{l} 3LiFePO_{4}+6NaOH=Fe_{3}O_{4}+NaLi_{2}PO_{4}+LiNa_{5}{\left(PO_{4}\right)}_{2}+H_{2}(g)+\\ 2H_{2}O(g) \end{array}$$

When Na_2_CO_3_ is used as an additive, the lithium recovery rate is 99.2%, and iron is recovered in elemental form; whereas when NaOH is used as an additive, the lithium recovery rate is 92.7%, and iron is recovered in the form of Fe_3_O_4_. When Na_2_CO_3_ is used, the lithium recovery rate is higher, and the produced iron is recovered in elemental form. This implies that the recovered iron is easy to separate and utilize, facilitating subsequent processing or utilization. Compared to the research by Zhang et al. [107], this method is safer and more effective. However, it generates a greater variety of lithium compounds, which may pose some difficulties in subsequent recovery processes.

Although the above-mentioned studies achieve efficient lithium extraction, the calcination temperature still needs to be maintained at a high level (≥600 °C), and the calcination time is relatively long. In response to this situation, Qu et al. [91] mixed (NH_4_)_2_SO_4_ with waste LFP cathode powder and calcined it in an air atmosphere. 

They accomplished rapid lithium extraction from LiFePO_4_ in just 5 min at 300 °C, converting LiFePO_4_ into FePO_4_ in an air atmosphere while Li transformed into soluble Li_2_SO_4_. The recovery rates were 97.8% for Li and 1.79% for Fe. In this process, both (NH_4_)_2_SO_4_ and O_2_ serve as oxidants. The reactions that occur are shown in Equation (26):

(26) $$\begin{array}{l} 2LiFePO_{4}+0.5O_{2}(g)+{\left(NH_{4}\right)}_{2}SO_{4}=Li_{2}SO_{4}+2FePO_{4}+2NH_{3}(g)+\\ H_{2}O(g) \end{array}$$

This method achieves rapid lithium extraction at moderate reaction temperatures, while the reaction products are easily recoverable. The reagents required for the reaction are inexpensive. This paper considers it as an important reference for industrial pyrometallurgical recovery of elements in LFP. The approximate pyrometallurgical process for LFP cathode materials can be referenced in Figure 8b.

## 5. Hydrometallurgical Process

Hydrometallurgical recovery processes are currently the most extensively researched methods for metal recovery from spent LIBs. This chapter divides it into two parts: (1) the leaching of valuable metals; (2) the separation and purification of the leached metals. 

### 5.1. Leaching Process

The leaching process involves reacting relevant leaching agents with spent cathode materials, converting valuable metals into solution form, and facilitating subsequent separation and extraction. In this paper, leaching processes are categorized into acid leaching, ammonia leaching, and bioleaching based on different leaching agents. 

#### 5.1.1. Acid Leaching

In spent lithium battery recycling, acid leaching involves dissolving valuable metal oxides into their ionic forms within an acidic environment, typically achieved through inorganic acids or organic acids, or their combinations. Common inorganic acids include H_2_SO_4_ [109], HCl [110], HNO_3_ [111], while common organic acids include citric acid [112], formic acid [113], acetic acid [114], and malic acid [115], etc. In general, the leaching efficiency of inorganic acids is superior to organic acids, but the strong acidity of inorganic acid waste liquids may corrode related experimental equipment. Additionally, inorganic acids can produce toxic gases such as NO_x_ and Cl_2_ during the leaching process. Therefore, compared to organic acids, more attention should be paid to handling the waste liquids and gases produced during leaching with inorganic acids. Since the positive electrode materials of spent LIBs have relatively stable structures, using organic acids or inorganic acids alone for leaching may result in incomplete reactions, thereby affecting the leaching efficiency. Therefore, current research typically adopts the combination of acid and reducing agent for metal leaching. H_2_O_2_ is the most common reducing agent [116], while other common reducing agents include glucose [117], ascorbic acid [118], and NaHSO_3_ [119], etc.

#### 5.1.2. Ammonia Leaching

Compared to traditional acid leaching methods, ammonia leaching strategies exhibit more refined ion selectivity [120]. In ammonia leaching, selective extraction of transition metals is achieved through the complexation of NH^4+^ ions from solutions such as ammonia water, ammonium carbonate, ammonium chloride, and ammonium sulfate with different metal ions [121]. The mechanism of this selective extraction is based on the differences in solubility and stability of the complexes formed between transition metals and ammonia ligands [122]. In contrast, acid leaching methods lack this precise selectivity and often result in the simultaneous extraction of various metal ions, increasing the difficulty of subsequent separation and purification. The advantages of ammonia leaching technology lie not only in its selectivity but also in its environmental friendliness and process sustainability. Compared to acid leaching, the waste liquid produced in ammonia leaching does not contain harmful acidic substances, reducing environmental pollution. Additionally, the solvents and reagents used in ammonia leaching are relatively mild [123]. It does not cause severe corrosion to equipment and pipelines, promoting the long-term stable operation of the equipment.

#### 5.1.3. Bioleaching

Bioleaching of spent lithium batteries utilizes the enzymatic systems and metabolic pathways of microorganisms to facilitate the dissolution and extraction of metals from used batteries by producing acids, lowering the environmental pH, reducing metals, and forming metal complexes [124]. This eco-friendly process can efficiently recover metal resources while reducing energy consumption, greenhouse gas emissions, and pollutant production. It has lower operational costs and processing hazards, making it more environmentally friendly compared to traditional pyrometallurgical and hydrometallurgical processes [125].

The microorganisms used can be divided into two categories: bacteria and fungi. The bacteria can be either mesophilic S/Fe-oxidizing bacteria or thermophilic S/Fe-oxidizing bacteria. The former utilizes atmospheric carbon dioxide as a carbon source and ferrous iron or elemental sulfur as primary energy sources. The microorganisms produce biogenic sulfuric acid and iron ions, thereby promoting metal dissolution and extraction [126]. Roy et al. [124] consider that bacteria capable of thriving at higher temperatures are advantageous for bioleaching, as elevated temperatures improve reaction kinetics. They propose that extreme thermophiles have higher metal bioleaching rates compared to moderate thermophiles and mesophiles due to their ability to grow at such high temperatures.

#### 5.1.4. Deep Eutectic Solvents Leaching

Deep eutectic solvents (DESs) are low-melting eutectic mixtures synthesized from 2 or 3 compounds through hydrogen bonding. Due to their simple preparation, wide availability, biodegradability, and exceptional ability to dissolve metal oxides, they have been widely applied in the field of spent LIB recycling since 2019 [127]. Most DESs possess reducing properties, meaning they can serve both as leaching agents and reducing agents. Current reports indicate that the metal leaching process using DESs generally involves reducing high-valence metals, forming metal complexes, and reacting metal oxides with DESs to generate water molecules.

In current recycling research, DESs can be divided into binary and ternary DESs. Typically, binary DESs are composed of a hydrogen bond donor (HBD) and a hydrogen bond acceptor (HBA) [128]. Due to their excellent ability to dissolve metal oxides, they are widely used in metal extraction and recovery, particularly in the recycling of spent LIBs. For example, Tian et al. [129] presented a DES made of ethylene glycol and hydroxylamine hydrochloride, achieving high leaching efficiencies for lithium and cobalt from LiCoO_2_ with notable solubility and sustainable recovery processes. Binary DESs exhibit excellent performance in chemical reaction kinetics and mass transfer capabilities; however, they often require longer leaching times. To address this issue, some studies have explored the use of ternary DESs, which can potentially enhance leaching efficiency and reduce processing time [130,131,132].

From an economic and sustainability perspective, using DESs to recycle cathode materials from spent lithium-ion batteries is highly significant. However, the complexity of their composition and the difficulty in controlling their properties present challenges, leading to higher costs. Despite these issues, optimizing DES formulations can potentially improve the cost-effectiveness and feasibility of this recycling method.

### 5.2. Methods of Enhanced Leaching

Emerging technologies, such as ultrasound and microwave treatment, can effectively promote the separation of cathode material and aluminum foil. Ultrasound technology uses high-frequency sound waves to create cavitation bubbles in a liquid. When these bubbles collapse, they generate localized high temperatures and pressures, disrupting the adhesive bonds between the cathode materials and the aluminum foil [133]. This method helps detach active materials without using harsh chemicals or high temperatures. Additionally, ultrasonic methods can also improve the efficiency of metal recovery and the performance of cathode regeneration. In the process of assisted leaching, the mechanical effects induced by ultrasound, such as micro-jets and shock waves, can cause micro-scale turbulence in the liquid and high-velocity collisions between solid particles [134,135]. Meanwhile, ultrasound prevents agglomeration through cavitation and mechanical effects, reducing particle size, improving diffusion, and lowering external mass transfer resistance through the product layer [136,137].

When performing high-temperature separations, some studies have utilized microwave heating to reduce roasting during pretreatment to enhance the water and acid leaching of precious metals from spent LIBs [138,139]. The use of microwave assistance in the extraction process can accelerate the heating process caused by ion collision in the leaching solution. Compared with the traditional extraction process, this method has many advantages, including reducing the heating time and the amount of solvent to improve the extraction efficiency [140]. Shih et al. [141] developed an optimized process using microwave-assisted acidic leaching, oxidative precipitation, and solvent extraction to efficiently recover and purify valuable metals from spent LIBs. Microwave assistance can also be used in hydrothermal regeneration of cathode electrodes, which is mentioned in Section 6.3.

In the pretreatment process, mechanical activation can also enhance leaching efficiency. This method involves using mechanical forces to increase the reactivity of the materials by reducing particle size, creating fresh surfaces, and inducing structural defects. Guan et al. [110] highlighted the importance of mechanical activation in enhancing Co extraction by reducing particle sizes, increasing specific surface area, and altering crystal structures, as confirmed by XRD and SEM analyses. Efficient Co leaching was achieved, with significant changes in the valence state of Co observed through XPS analysis. The novel mechanochemical process achieved high leaching ratios for Li, Co, Mn, and Ni from waste LIBs, underscoring its significance for metal recovery.

### 5.3. Separation and Purification

The leachate after leaching contains various valuable metal elements but also a large amount of impurities and other metal components. Therefore, it is necessary to further separate and purify the leachate to extract the target metals and purify the product. This process involves various physical and chemical methods, such as solvent extraction and ion exchange, aimed at achieving effective metal separation and purification, laying the foundation for subsequent metal recovery and reuse. This paper summarizes the separation and purification processes as precipitation, organic extraction, ion exchange, and electrochemical methods.

#### 5.3.1. Precipitation Method

The precipitation method involves selecting an appropriate precipitant after leaching the cathode materials of spent LIBs to react with metal ions in the leachate, thereby achieving metal separation and extraction. In hydrometallurgical processes, Na_2_CO_3_ is commonly used for selective lithium recovery. When Na_2_CO_3_ is chosen as the precipitant, lithium will be recovered in the form of Li_2_CO_3_. For instance, Jha et al. [142] used Na_2_CO_3_ as the precipitant, maintained the solution pH between 11 and 12 using sodium hydroxide and a diluent, filtered the Li precipitate product, and removed impurities with distilled washing water, obtaining Li_2_CO_3_ product. The recovery products of other metals are explained in Chapter Five and will not be individually listed here.

The precipitation method facilitates the effective separation of different metal components in spent batteries, providing crucial preliminary treatment for subsequent metal recovery processes. It is easily scalable for industrialization and can handle relatively large quantities. However, the key lies in finding suitable precipitants, and its product purity is relatively lower compared to other methods, necessitating further process optimization to improve product purity. 

#### 5.3.2. Organic Solvent Extraction

Organic solvent extraction is an effective method widely used for metal separation and purification in hydrometallurgical recovery processes [143]. This technique is based on the differences in interaction between various metal ions and organic solvents to achieve metal separation. It primarily relies on one or more organic solvents (referred to as extractants) capable of selectively binding with target metal ions. In this process, the aqueous phase containing metal ions is mixed with the organic phase containing added extractants. Due to the affinity between metal ions and extractants, some metal ions transfer from the aqueous phase to the organic phase.

Commonly used extractants include trioctylamine (TOA) [144], D2EHPA [145], Cyanex 272 [146], TBP [145], etc. In the extraction process of spent LIBs, compared to the use of a single extractant, a combination of two or more extractants is often employed to enhance the selectivity of metal ions through synergistic effects. This mixed extraction strategy not only enhances extraction efficiency but also significantly improves the separation between target metals and impurities by optimizing the proportions and conditions of different extractants. Sen et al. [147] developed a mixed solvent extraction system specifically designed to effectively separate and purify lithium from waste solutions containing lithium in the recycling process of LIBs. By using a combination of D2EHPA and TBP, along with kerosene as a diluent, this system demonstrated high selectivity for lithium extraction under optimized conditions. This method not only improves lithium recovery efficiency to over 95% but also effectively retains sodium ions in the raffinate during the extraction process, thereby reducing sodium impurities in the lithium product.

#### 5.3.3. Electrochemical Method

In the hydrometallurgical recovery process of spent LIBs, electrochemical methods are crucial for metal separation and purification. This method utilizes electrochemical reactions during electrolysis to recover and purify metal ions in the leachate. Electrochemical methods are typically conducted in electrolytic cells containing an anode and a cathode. The anode is commonly made of inert metals or other materials, while the cathode uses high-purity metals or other conductive materials. The leachate containing metal ions is introduced into the electrolytic cell. Metal ions in the leachate migrate toward the cathode or anode under the influence of the electric field. Prabaharan et al. [148] extracted Co and Mn from ternary cathode materials through sulfuric acid leaching and electrochemical deposition, achieving recovery rates of 97% and 99%, respectively. Electrochemical methods are highly promising for the recovery of metals from spent LIBs and other electronic waste, with the potential to further drive the circular economy and resource sustainability. However, the costs of electrolysis equipment and power sources are relatively high, and precise control of electrolyte composition and concentration is required to avoid secondary pollution and ensure metal deposition efficiency.

#### 5.3.4. Ion Exchange Method

The ion exchange method is a technique that utilizes the selective adsorption properties of ion exchange resins or media to separate and recover metal ions. During the ion exchange process, various metal ions pass through a column filled with ion exchange resin, where the target metal ions are adsorbed onto the resin. Subsequently, these metal ions are released from the resin and recovered using an eluent. Chiu et al. [149] employed Dowex M4195 resin as the separation method, eliminating the need for additional impurity removal operations. After loading and elution, three different grades were obtained: a 99.0% nickel concentrate, a 98.5% cobalt concentrate, and a lithium/manganese-rich concentrate, allowing the separation of nickel, cobalt, and manganese.

#### 5.3.5. Ionic Liquids Extraction

Ionic liquids (ILs) are ionic compounds composed of specific cations and anions that exist in a liquid state near room temperature. Due to the wide variety of selectable cations and anions, ionic liquids are highly designable. With appropriate design, ionic liquids can exhibit advantages such as low volatility, high stability, a wide liquid range, high conductivity, and high solubility. Consequently, they are widely regarded in fields such as catalysis, separation, and batteries [150,151,152]. Due to ILs’ wide variety, they can be used as leaching agents and extractants in hydrometallurgical processes. When ionic liquids (ILs) are used in leaching processes, they need to possess a certain degree of reducing ability [153]. Currently, there is limited research in this area, and it is not yet possible to confirm their economic viability as leaching agents.

ILs can be applied to aqueous solvent extraction. Unlike traditional aqueous solvent extraction, this method utilizes water-immiscible organic ionic liquids. These ionic liquids selectively remove target metal ions from the aqueous solution, forming complexes with them and facilitating their transfer between phases. This method can preferentially extract Li, as well as divalent metal ions such as Ni, Co, and Mn [154,155,156]. Non-aqueous solvent extraction is presently less prevalent in application compared to aqueous solvent extraction. In this technique, DESs are used as leaching agents, and ILs are then employed to extract metal ions from leachate containing less than 50 vol% water [157].

#### 5.3.6. Different Leaching Processes’ Purification Methods

After acid leaching, there are several methods available for metal separation and purification, including precipitation, organic solvent extraction, electrochemical deposition, ion exchange, and ionic liquids extraction. Among these, the most widely used method is precipitation [142,148,158,159,160]. Unlike acid leaching, ammonia leaching does not require the separation and purification methods mentioned in Section 5.3. In the ammonia leaching system, metal ions in the solution combine with ammonia and other ligands (such as sulfate ions, sulfite ions, etc.) to form stable double salts. These double salts precipitate from the solution through methods such as evaporation crystallization, achieving the purpose of metal recovery [161,162].

Currently, most research on bioleaching focuses only on the metal dissolution stage, with little exploration of subsequent product recovery. Some studies have used chemical precipitation methods to recover Li and Co from fungal leachates [163]. Biswal et al. [164] suppose that biotechnological methods such as bioprecipitation, biosorption, and bioelectrochemical systems (e.g., microbial fuel cells and microbial electrolysis cells) can be used for metal recovery. DESs can effectively separate Ni and Co. After leaching, lithium and these valuable metals can be recovered using chemical precipitation, organic solvent extraction, and ion solution extraction [157]. Additionally, some studies suggest that adding certain reagents, which can act as hydrogen bond donors (HBD) or hydrogen bond acceptors (HBA) without affecting the initial DES, is a promising approach [165]. However, there is currently insufficient literature to support this claim comprehensively. It is important to note that, as with other leaching methods, if solid products are desired after solvent/solution extraction, precipitation treatment is still required [160].

### 5.4. Hydrometallurgy of Nickel–Cobalt–Manganese Cathode Materials

**Figure 9:**
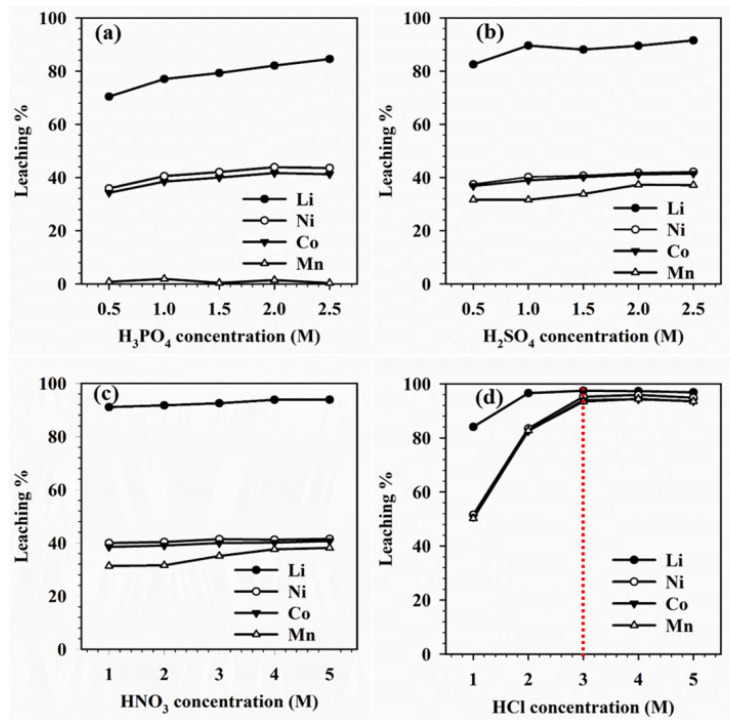
(**a**) H_3_PO_4_, (**b**) H_2_SO_4_, (**c**) HNO_3_, and (**d**) HCl—different inorganic acids affect the leaching efficiency of NCM622 cathode materials for Li, Ni, Co, and Mn (The red line represents the HCL concentration with the best effect) [166], Elsevier.

Inorganic acid leaching is a commonly used method for extracting metals from nickel–cobalt–manganese cathodes, and different types of inorganic acids can affect the leaching efficiency of metals (as shown in Figure 9).

**Figure 10:**
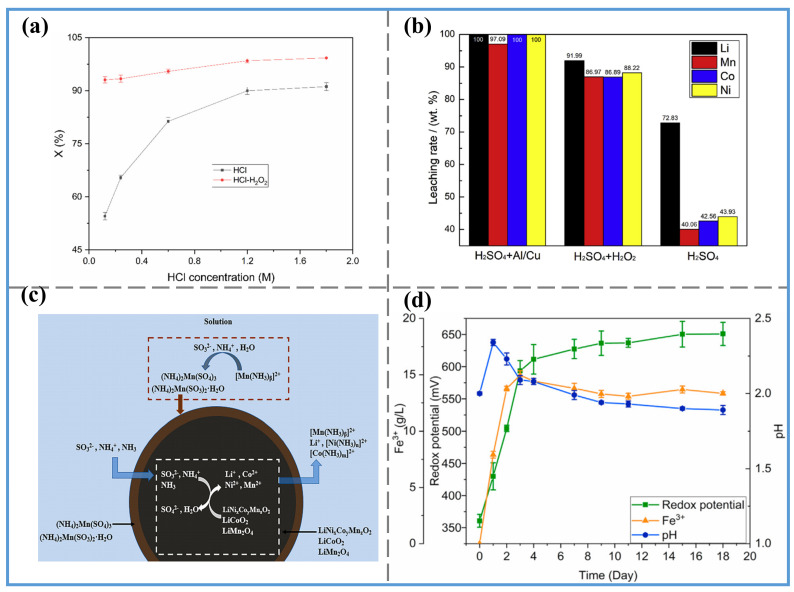
(**a**) Effect of hydrochloric acid concentration (with or without H_2_O_2_) on sample dissolution [170], MDPI; (**b**) Leaching rate of transition metals by different methods (especially using aluminum powder and copper powder as analytical reagents) [174], Elsevier; (**c**) Schematic diagram of selective recovery of valuable metals from waste LIB by ammonia leaching [161], Taylor and Franics; (**d**) Changes in pH, redox potential, and Fe^3+^ concentration of A. ferrooxidans growth curve [183], Elsevier.

Current research indicates that adding a reducing agent during the leaching process can enhance leaching efficiency, with H_2_O_2_ being the most commonly used reducing agent. For instance, in the study by Promphan [167], the extraction rates of nickel, manganese, and cobalt were less than 60% without H_2_O_2_; when this reducing agent was used, the extraction rates of these metals exceeded 90%. The reason is reducing as much Co^3+^ to Co^2+^ and Mn^4+^ to Mn^2+^ as possible helps these metals dissolve more easily. However, Guimarães et al. [168] argue that reducing agents are not essential for improving leaching efficiency. They achieved high-efficiency leaching by using a two-step grinding method with a Willey mill before leaching. Since their study did not include a control group with added reducing agents, it is unclear whether adding a reducing agent in the original experiment would have improved leaching efficiency. Reducing agents can not only improve leaching efficiency but also reduce the amount of acid needed for leaching. HCl is considered the most suitable inorganic acid for leaching LCO, but it necessitates a substantial amount of acid during the leaching process [169]. To address this issue, Drajlin et al. [170] compared the dissolution of LCO in an HCl medium with and without H_2_O_2_ (as shown in Figure 10a). When LCO reacts with HCl alone, the chemical equation of the reaction is shown in Equation (27):

(27) $$2LiCoO_{2(s)}+8HCl_{\left(aq\right)}\to 2CoCl_{2(aq)}+Cl_{2(g)}+2LiCl_{\left(aq\right)}+4H_{2}O$$

When LCO reacts with HCl and H_2_O_2_, the chemical equation of the reaction is shown in Equation (28):

(28) $$\begin{array}{l} 2LiCoO_{2(s)}+2H_{2}O_{2(aq)}+8HCl_{\left(aq\right)}\to 2CoCl_{2(aq)}+Cl_{2(g)}+2LiCl_{\left(aq\right)}+O_{2(g)}\\ +6H_{2}O \end{array}$$

The results indicate that using 1.8 M HCl at 348 K for 60 min achieved the highest solubility of LCO, which was 91.0%. Additionally, in the reducing medium, a slightly higher oxide solubility (93.0%) was achieved at the same temperature in half the time, with an HCl concentration more than ten times lower. In recent years, there have been few studies on HNO_3_ leaching of spent LIBs, and recent research also indicates that using nitric acid to leach nickel–cobalt–manganese cathode materials is not effective [171]; therefore, this will not be further discussed in this paper. H_2_SO_4_ is cheaper compared to HCl and HNO_3_, and there is more research on its use for the recovery of spent nickel–cobalt–manganese cathode materials. Chen et al. [172] leached LCO and NCM cathode materials using 2 M H_2_SO_4_ and 10 vol% H_2_O_2_ at 70 °C and 300 rpm, with a liquid–solid mass ratio of 30 mL/g. The leaching reactions are shown in Equations (29)–(31):

(29) $$6{\mathrm{LiNi}}_{\frac{1}{3}}{\mathrm{Mn}}_{\frac{1}{3}}{\mathrm{Co}}_{\frac{1}{3}}O_{2(s)}+9H_{2}{\mathrm{SO}}_{4(\mathrm{aq})}+H_{2}O_{2(\mathrm{aq})}\to 2{\mathrm{MnSO}}_{4(\mathrm{aq})}+2{\mathrm{NiSO}}_{4(\mathrm{aq})}$$

(30) $$\begin{array}{l} 2{\mathrm{LiCoO}}_{2(s)}+3H_{2}{\mathrm{SO}}_{4(\mathrm{aq})}+H_{2}O_{2(\mathrm{aq})}\to 2{\mathrm{CoS}O}_{4(\mathrm{aq})}+{\mathrm{Li}}_{2}{\mathrm{SO}}_{4(\mathrm{aq})}+4H_{2}O_{\left(g\right)}+\\ O_{2(g)} \end{array}$$

(31) $$\begin{array}{c} \begin{array}{l} 6LiNi_{\frac{1}{3}}Mn_{\frac{1}{3}}Co_{\frac{1}{3}}O_{2(s)}+9H_{2}SO_{4(aq)}+H_{2}O_{2(aq)}\to \\ 2MnSO_{4(aq)}+2NiSO_{4(aq)}+2CoSO_{4(aq)}+3Li_{2}SO_{4(aq)}+10H_{2}O_{\left(g\right)}+2O_{2} \end{array} \end{array}$$

After leaching, Co and Mn were separated using D2EHPA to obtain high-purity Co. Next, Ni was selectively precipitated with DMG, forming a solid complex. Finally, during the chemical precipitation process, the remaining Li in the leachate was recovered as Li_2_CO_3_ using saturated Na_2_CO_3_, while Co, Mn, and Ni were recovered as hydroxides using NaOH. The purity of the resulting cobalt, nickel, and lithium products exceeded 99.5%, and the manganese products had a purity of over 90%. In addition to the extra reducing agent, Peng et al. [173] discovered that Cu, Al, and Fe in waste lithium batteries can significantly enhance the leaching efficiency of Li and Co, achieving nearly 100%. However, they did not account for the precise amounts of Cu and Al foil used, nor did they detail the corresponding purification methods. Based on this problem, Yu et al. [174] determined the feasibility and theoretical requirements of Al and Cu through simulation experiments and thermodynamic analysis (Figure 10b). Subsequently, they revealed the optimal leaching conditions through lightly screened practical cumulative effect experiments. Using an appropriate amount of H_2_SO_4_ to leach the NCM cathode material and Cu/Al foil mixture, NaOH solution was added to adjust the pH multiple times and filter. Ultimately, nearly 100% of the valuable metal elements in the powder dissolved into the aqueous solution, eventually converting to CuO, NaAlCO_3_(OH)_2_, NiCoMn(OH)_x_ precursor, and Li_2_CO_3_.

Most of the inorganic acids used in inorganic acid leaching are strong acids, which are highly corrosive and release Cl_2_, SO_3_, and NO_x_ during the leaching process. These factors need to be carefully considered during industrialization. Compared to inorganic acids, organic acids can leach nickel–cobalt–manganese cathodes under milder conditions. Demarco et al. [175] compared the leaching effects of three organic acids—malic acid, citric acid, and formic acid—combined with H_2_O_2_ on NCM and LCO. The reactions of LCO with malic acid are shown in Equations (32) and (33):

(32) $$\begin{array}{c} \begin{array}{l} 2LiCoO_{2}(s)+6C_{4}H_{6}O_{5}(aq)+H_{2}O_{2}\to \\ 4LiC_{4}H_{5}O_{5}(aq)+2Co{\left(C_{4}H_{5}O_{5}\right)}_{2}(aq)+4H_{2}O(l)+O_{2}(q) \end{array} \end{array}$$

(33) $$\begin{array}{l} 2LiCoO_{2}(s)+6C_{4}H_{5}O_{5}^{-}(aq)+2Li^{+}(aq)+2Co^{2+}(aq)+H_{2}O_{2}\to \\ 2Li_{2}C_{4}H_{4}O_{5}(aq)+4CoC_{4}H_{4}O_{5}(aq)+4H_{2}O(l)+O_{2}(g) \end{array}$$

The reactions of LCO with citric acid are shown in Equations (34)–(36):

(34) $$\begin{array}{l} 6H_{3}Cit(aq)+2LiCoO_{2}(s)+H_{2}O_{2}\left(aq\right)=\\ 2Li^{+}(aq)+6H_{2}Cit^{-}(aq)+2Co^{2+}(aq)+4H_{2}O+O_{2}(g) \end{array}$$

(35) $$\begin{array}{l} 6H_{2}Cit^{-}(aq)+2LiCoO_{2}(s)+H_{2}O_{2}(aq)=\\ 2Li^{+}(aq)+2Co^{2+}(aq)+6HCit^{2-}(aq)+4H_{2}O+O_{2}(g) \end{array}$$

(36) $$\begin{array}{l} 6HCit^{2-}(aq)+2LiCoO_{2}(s)+H_{2}O_{2}(aq)=\\ 2Li^{+}(aq)+2Co^{2+}(aq)+6Cit^{3-}(aq)+4H_{2}O+O_{2}(g) \end{array}$$

The reactions of NCM333 with formic acid are shown in Equations (37) and (38):

(37) $$2Al(s)+6HCOOH(aq)\to 2C_{3}H_{3}AlO_{6}(aq)+3H_{2}(g)$$

(38) $$\begin{array}{l} 6LiNi_{\frac{1}{2}}Co_{\frac{1}{2}}Mn_{\frac{1}{2}}O_{2}(s)+21HCOOH(aq)\to \\ 2C_{2}H_{2}NiO_{4}(aq)+2C_{2}H_{2}CoO_{4}(aq)+2C_{2}H_{2}MnO_{4}(aq)+6CHLiO_{2}(aq) \end{array}$$

The results show that among these three organic acids, DL-malic acid has the best leaching effect on NCM cathode materials. By employing 2 M DL-malic acid, 6% (*v*/*v*) H_2_O_2_, with a solid-to-liquid ratio of 1:20 (*m*/*v*), at 95 °C for a leaching duration of 60 min, over 90% of Co, Li, and Mn can be successfully leached. Most organic acids require the addition of reducing agents such as H_2_O_2_ or glucose to improve leaching efficiency. However, ascorbic acid is different because of its reducing properties, and it is used in combination with various leaching agents to enhance leaching efficiency, such as sulfuric acid [176], citric acid [177], and tartaric acid [178]. Given its reducing properties and certain leaching abilities, Li et al. [179] achieved ultra-fast leaching of Li and Co using 1.25 mol/L ascorbic acid at 70 °C. In this process, ascorbic acid dissolves waste LiCoO_2_ to form soluble C_6_H_6_O_6_Li_2_, while Co^3+^ in LiCoO_2_ is further reduced by ascorbic acid to soluble Co^2+^ During this period, ascorbic acid (C_6_H_8_O_6_) is oxidized to dehydroascorbic acid (C_6_H_6_O_6_). The reactions that occur is shown in Equation (39):

(39) $$4C_{6}H_{8}O_{6}+2LiCoO_{2}=C_{6}H_{6}O_{6}+C_{6}H_{6}O_{6}Li_{2}+2C_{6}H_{6}O_{6}Co+4H_{2}O$$

Ultimately, the leaching rate of Co reached 94.8%, and the leaching rate of Li reached 98.51%. Compared to other organic acids, the uniqueness of ascorbic acid lies in its reducing properties coupled with a certain leaching ability. This not only accelerates the reaction rate but also effectively disposes of the waste and reduces the adverse impact on the environment in the process of metal ion leaching. Therefore, ascorbic acid has broad application prospects in the recycling of waste battery materials, providing new ideas and methods for related research.

Using ammonia leaching to extract nickel–cobalt–manganese cathode materials is also a recent research focus. Ammonia leaching selectively separates Mn and Al from Ni, Co, and Li during the leaching process, streamlining the subsequent metal ion separation and recovery process. In the ammonia leaching of nickel–cobalt–manganese cathode materials, typically, multiple ammonia-based reagents, including reducing agents, are employed. [122,161,162]. Wang et al. [162] used ammonia (NH_3_) and ammonium sulfate ((NH_4_)_2_SO_4_) as leaching agents, and sodium sulfite (Na_2_SO_3_) as a reducing agent to leach Li, Ni, Co, and Mn from spent NCM523. The reaction equations are shown in Equations (40)–(45):

(40) $$Ni^{2+}+n_{1}NH_{3}=Ni{\left(NH_{3}\right)}^{2+}$$

(41) $$Co^{2+}+n_{2}NH_{3}=Co{\left(NH_{3}\right)}_{n2}^{2+}$$

(42) $$Li^{+}+n_{3}NH_{3}=Li{\left(NH_{3}\right)}_{n3}^{+}$$

(43) $$Mn^{2+}+n_{4}NH_{3}=Mn{\left(NH_{3}\right)}_{n4}^{2+}$$

(44) $$H_{2}O+2Co^{3+}+SO_{3}^{2-}=SO_{4}^{2-}+2Co^{2+}+2H^{+}$$

(45) $$Mn^{2+}+2NH_{4}^{+}+2SO_{3}^{2-}+H_{2}O={\left(NH_{4}\right)}_{2}Mn{\left(SO_{3}\right)}_{2}\cdot H_{2}O_{\left(S\right)}$$

The dissolved Ni, Co, Li, and Mn from NCM523 can be leached together with ammonia as coordination complexes, with Mn subsequently precipitating as an inclusion compound.

With (NH_4_)_2_SO_4_ concentration at 1.5 mol/L, NH_3_ concentration at 4 mol/L, a solid–liquid ratio of 10:1, Na_2_SO_3_ concentration at 0.5 mol/L, leaching time set to 180 min, and reaction temperature maintained at 90 °C, the leaching efficiencies were as follows: Li at 96.2%, Co at 89.9%, Ni at 90.1%, and Mn at 9.2%. It indicates that the ammonia leaching method can selectively leach Ni, Co, and Li without leaching Mn. The manganese(II) in the leaching residue precipitates as (NH_4_)_2_Mn(SO_3_)_2_⋅H_2_O, which facilitates further separation and purification. Similarly, Liu et al. [161] used (NH_3_)_2_SO_3_, NH_3_·H_2_O, and (NH_3_)_2_CO_3_ to leach mixed powders of LCO, NCM, and LMO. The leaching process is shown in Figure 10c. Under optimal conditions, the leaching rates of Co, Ni, Li, Mn, and Al were 84.56%, 64.13%, 90.31%, 4.53%, and 1.72%, respectively. Ammonia leaching also presents certain challenges, as its process conditions need to be relatively strictly controlled. Parameters such as NH4+ ion concentration, temperature, and pH value in the solution must be precisely controlled to ensure the effectiveness and stability of selective extraction. From the listed studies, it can be seen that the efficiency of metal leaching by ammonia is lower than that of acid leaching. This is because the solubility and stability of transition metal complexes are influenced by various factors. In-depth research on the complexation characteristics of different metal ions is needed to optimize the extraction process and improve extraction efficiency and purity.

**Figure 11:**
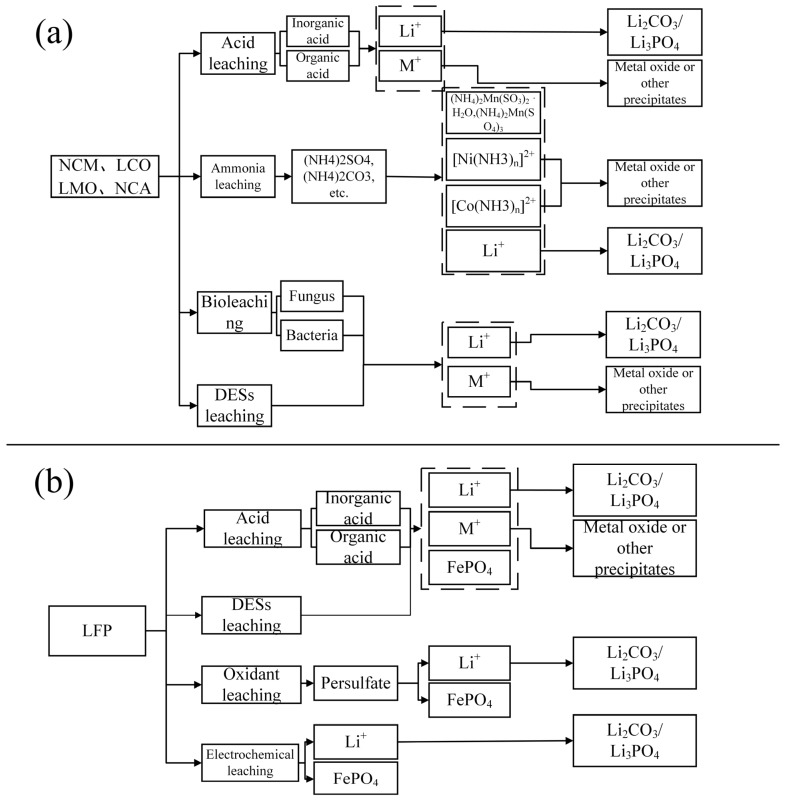
(**a**) The process of wet metal extraction from NCM cathode materials; (**b**) The process of wet metal extraction from LFP cathode materials.

In the field of bioleaching, Jegan et al. [180] achieved optimal leaching of valuable metals from LCO using the autotrophic bacterium Acidithiobacillus ferrooxidans. The changes in pH, redox potential, and Fe^3+^ concentration in the growth curve of A. ferrooxidans are shown in Figure 10d. The study shows that under optimized conditions, this method can efficiently recover various precious metals. Do et al. [181] used Acidithiobacillus ferrooxidans to bioleach spent ternary lithium-ion batteries, achieving leaching efficiencies of 85.5% for Ni, 91.8% for Mn, 90.4% for Co, and 89.9% for Li within 6 h. Additionally, they successfully removed impurities such as copper, aluminum, and iron through air oxidation and pH adjustment, thereby avoiding the difficulties of separating and purifying impurity elements from the leachate. It indicates that cost-effective and environmentally friendly bioleaching technology has potential in the recycling of spent LIBs. However, bioleaching faces challenges in environments rich in heavy metals because bacteria struggle to reproduce and survive under such conditions. The kinetics of the bioleaching process are slow, and it usually can only be carried out at low solid–liquid ratios, further limiting its industrial application and efficiency. To overcome these limitations, further research and development of improved bacterial strains and optimized process conditions are needed to enhance the efficiency and applicability of bioleaching. The hydrometallurgical process for nickel–cobalt–manganese cathode materials can be referred to in Figure 11a. In recent years, DESs have been increasingly used for nickel–cobalt–manganese (NCM) cathode materials. For instance, He et al. [182] introduced a new deep eutectic solvent (DES) made of choline chloride and phenylphosphinic acid for leaching metals (Li, Ni, Co, and Mn) from spent NCM battery cathodes. The DES effectively leached these metals under mild conditions, achieving efficiencies of 97.7% for Li, 97.0% for Co, 96.4% for Ni, and 93.0% for Mn at 100 °C in 80 min with a liquid–solid ratio of 90 mL/g. The leaching followed the logarithmic law equation, controlled by a chemical reaction, and Ni and Co were reduced to lower valences in the solution.

### 5.5. Hydrometallurgy of LFP Cathode Materials

Unlike nickel–cobalt–manganese cathode materials, the most valuable element for recycling in LFP is Li, leading some studies to focus solely on the leaching and recovery of lithium when opting for the hydrometallurgical recycling of LFP. In the selective leaching of lithium from LFP using inorganic acids, an oxidizing agent is commonly employed. This agent facilitates the oxidation of LFP material to FePO_4_, allowing Li^+^ ions to diffuse into the acidic solution, thereby achieving selective leaching. Jin et al. [184] used air as the oxidizing agent and H_2_SO_4_ as the leaching agent to accomplish the selective leaching of lithium, with the core reaction shown in Equation (46):

(46) $$4LiFePO_{4}+O_{2}(air)+4H^{+}=4Li^{+}+2H_{2}O+4FePO_{4}$$

The results indicate that in the lithium leaching process, the oxidation of Fe and the deintercalation of Li transpire simultaneously, leading to a gradual increase in the ratio of FePO_4_ to LiFePO_4_ phases, while the olivine structure remains predominantly unaffected. A remarkable 99.3% of Li is leached out, with only 0.02% of Fe and P dissolved. Li+ is subsequently recovered as Li_2_CO_3_ powder using Na_2_CO_3_.In the previously discussed acid leaching of nickel–cobalt–manganese cathodes, H_2_O_2_ was used as a reducing agent to assist leaching; however, in the selective acid leaching of LFP for Li extraction, it can serve as an oxidizing agent. Li et al. [185] conducted leaching in 0.3 M H_2_SO_4_ with an H_2_O_2_/Li molar ratio of 2.07 and H_2_SO_4_/Li molar ratio of 0.57, at 60 °C for 120 min, achieving leaching rates of 96.85% for Li and 0.027% for Fe. Subsequently, Na_3_PO_4_ is introduced as a precipitant to recover Li from the solution, with approximately 95.56% of Li precipitated and recovered as Li3PO4. Additionally, FePO_4_ in the leaching residue is directly recovered by burning off the carbon residue at 600 °C for 4 h. The leaching reactions are shown in Equations (47) and (48):

(47) $$2LiFePO_{4}+H_{2}SO_{4}+H_{2}O_{2}\to Li_{2}SO_{4}+2FePO_{4}↓+2H_{2}O$$

(48) $$2Fe^{2+}+H_{2}O_{2}+2H^{+}\to 2Fe^{3+}+2H_{2}O$$

In addition to H_2_SO_4_, H_3_PO_4_ is also commonly used for the recovery of LFP. Using this method, Li_3_PO_4_ and FePO_4_ can be recovered from LFP, but Li_3_PO_4_ is not directly produced and requires pH adjustment to precipitate. Furthermore, certain preconditions must be met to activate the cathode material to enhance the leaching efficiency, making the use of H_3_PO_4_ for leaching LFP less economical than H_2_SO_4_ [186]. When using inorganic acids to separately extract Li, Fe, and P from LFP, it is usually necessary to use a reducing agent to prevent the oxidation of Fe^2+^ to Fe^3+^ during the acid-leaching process. Yang et al. [187] used sulfuric acid as the leaching agent and ascorbic acid as the reducing agent to extract Li, Fe, and P from LFP. The reactions involved are shown in Equation (49):

(49) $$2LiFePO_{4}+3H_{2}SO_{4}\to 2Li^{+}+2Fe^{2+}+3SO_{4}^{2-}+2PO_{4}^{3-}+6H^{+}$$

However, the separate extraction of Li, Fe, and P is of limited value, as subsequent recovery processes require further precipitation. When only extracting Li from LFP, Fe and P are recovered in the form of FePO_4_. FePO_4_ itself is a utilizable compound, which can be used for the solid-phase synthesis of LFP cathode, as will be mentioned later.

Similarly to inorganic acids, the selective extraction of Li using organic acid leaching also requires the assistance of an oxidizing agent. Yang et al. [188] used acetic acid and H_2_O_2_ to selectively extract lithium from LFP, and the reactions involved are shown in Equation (50):

(50) $$\begin{array}{c} LiFePO_{4}(II)(s)+CH_{3}COOH(aq)+\frac{1}{2}H_{2}O_{2}(aq)\\ \to FePO_{4}(III)(s)+CH_{3}COO^{-}(aq)+Li^{+}(aq)+H_{2}O(l) \end{array}$$

The dissolution recovery rate of Li is as high as over 95.05%, with leaching selectivity reaching around 94.08%. The purity of Li_2_CO_3_ after recovery with Na_2_CO_3_ can reach 99.95 wt%, meeting the battery-grade purity standard. However, Mahandra et al. [189] argued that acetic acid has a significantly higher chemical oxygen demand (COD) for wastewater, which greatly increases the cost of using acetic acid. Formic acid, on the other hand, has one-third the COD of acetic acid and is ten times stronger, making it an excellent reagent for organic acid leaching. In this context, the reactions involved in leaching waste LFP cathode materials using formic acid and H_2_O_2_ are shown in Equation (51):

(51) $$\begin{array}{c} LiFePO_{4}(s)+HCOOH(aq)+\frac{1}{2}H_{2}O_{2}(aq)\\ \to FePO_{4}(s)+HCOO^{-}(aq)+Li^{+}(aq)+H_{2}O(aq) \end{array}$$

Li+ is precipitated in situ using a saturated Na_3_PO_4_ solution at pH = 12.5 and 90 °C. The purity of the product reaches over 99.5%. The crystallization pH range of FePO_4_ is from 1.1 to 9.0, and at lower pH values, it will exist in the form of Fe^3+^ or Fe^2+^ [190]. Therefore, when using organic acids to leach all elements from LFP, it is necessary to use stronger acidic organic acids to separate iron and phosphorus. Currently, some scholars have used methanesulfonic acid to achieve the complete leaching of all elements from LFP [191]. Similar to inorganic acids, individually leaching Li, Fe, and P is not more economical than selectively leaching Li; therefore, this paper will not further elaborate on this topic.

The importance of acid leaching combined with an oxidizing agent for selective lithium extraction has already been emphasized in the text. In addition to using acid combined with an oxidizing agent for leaching, some peroxysulfate oxidants such as sodium persulfate (Na_2_S_2_O_8_) [192] and ammonium persulfate ((NH_4_)_2_S_2_O_8_) [193] can achieve selective lithium extraction with a single reagent. The reactions with LFP are shown in Equations (52) and (53), respectively:

(52) $$2LiFePO_{4}+Na_{2}S_{2}O_{8}=2FePO_{4}↓+Li_{2}SO_{4}+Na_{2}SO_{4}$$

(53) $$2LiFePO_{4}+{\left(NH_{4}\right)}_{2}S_{2}O_{8}\to 2FePO_{4}↓+Li_{2}SO_{4}+{\left(NH_{4}\right)}_{2}SO_{4}$$

The results show that with 1.05 times the theoretical amount of Na_2_S_2_O_8_, a solid–liquid ratio of 300 g·L^−1^, a temperature of 25 °C, and a duration of 20 min, the leaching rate of Li reaches 99.9%. Meanwhile, the leaching rates of Al, Fe, and P are 0.584%, 0.048%, and 0.387%, respectively. Good results were also obtained when using (NH_4_)_2_S_2_O_8_ for leaching. Although oxidants such as Na_2_S_2_O_8_ and (NH_4_)_2_S_2_O_8_ have stronger oxidation capabilities than commonly used H_2_O_2_ and O_2_, their prices are relatively higher. Therefore, the appropriate dosage is a key factor affecting economic feasibility. From current research, using air (or oxygen) as an oxidizing agent achieves the highest leaching rate for Li. Moreover, this oxidant is more readily available and easier to control in terms of dosage, thus presenting good prospects for industrialization.

**Figure 12:**
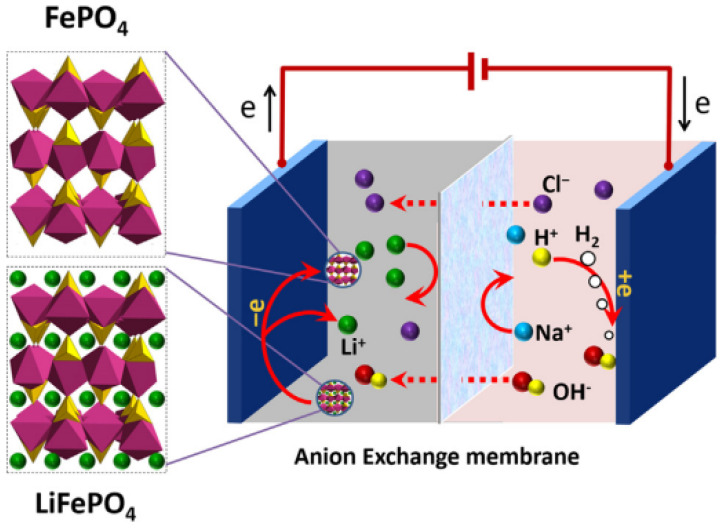
Schematic diagram of LFP electrolytic metallurgy [193], Elsevier.

In recent research, the electrolysis method has been regarded as a green selective lithium extraction method. Li et al. [194] proposed a novel approach that integrates the charging mechanism of LiFePO_4_ batteries with a slurry electrolysis process to selectively extract Li and FePO_4_/C from waste LiFePO_4_. Illustrated in Figure 12, a two-compartment electrolytic cell is utilized, with an anion exchange membrane facilitating the separation. NaCl serves as the supporting electrolyte. In the anode compartment, spent LiFePO_4_ powder undergoes oxidation to FePO_4_, akin to the charging process, thereby releasing Li into the electrolyte. Simultaneously, a hydrogen evolution reaction occurs at the cathode compartment, generating NaOH. The reactions at the positive and negative electrodes are shown, respectively, as Equations (54) and (55):

(54) $$LiFePO_{4}-e=Li^{+}+FePO_{4}$$

(55) $$2H_{2}O+2e=H_{2}↑+2OH^{-}$$

Under optimal process conditions, over 98% of Li is leached into the electrolyte, while more than 96% of Fe is recuperated as FePO_4_/C. The electrolysis method is a simple, green, and economically feasible process, but it is currently at the laboratory stage. The high investment and maintenance costs of equipment due to the use of corrosion-resistant materials for manufacturing electrolytic cells and electrodes are issues that need to be considered for industrialization.

In summary, both organic acid leaching and inorganic acid leaching can achieve excellent recovery efficiency when extracting organic elements from LFP. Although inorganic acids are more corrosive, their lower cost gives them greater potential for industrial application. Research on simultaneous leaching of all elements is limited because, although it can extract valuable components in one step, the separation process is complex and can easily result in lithium loss. After selective lithium leaching, the recovered solid powder can be directly used for the regeneration of LFP cathode materials. Therefore, in production practice, the selective leaching method can more effectively and economically recover valuable elements from LFP. To save reagents, recent studies have used mechanical activation-assisted acid leaching for selective lithium extraction from LFP [195,196,197], which could be considered for future industrial applications. The latest research shows that DESs can be applied for the leaching of lithium iron phosphate (LFP). For example, Chen et al. [198] used a natural deep eutectic solvent (NADES) composed of glucose and lactic acid, achieving a leaching efficiency of up to 96.5% for LFP under mild conditions. In this process, Li was selectively separated from Fe. After filtration, both Li and Fe were present as ions in the filtrate. More research is needed to demonstrate the feasibility of using deep eutectic solvents for LFP leaching.

## 6. Cathode Regeneration

**Figure 13:**
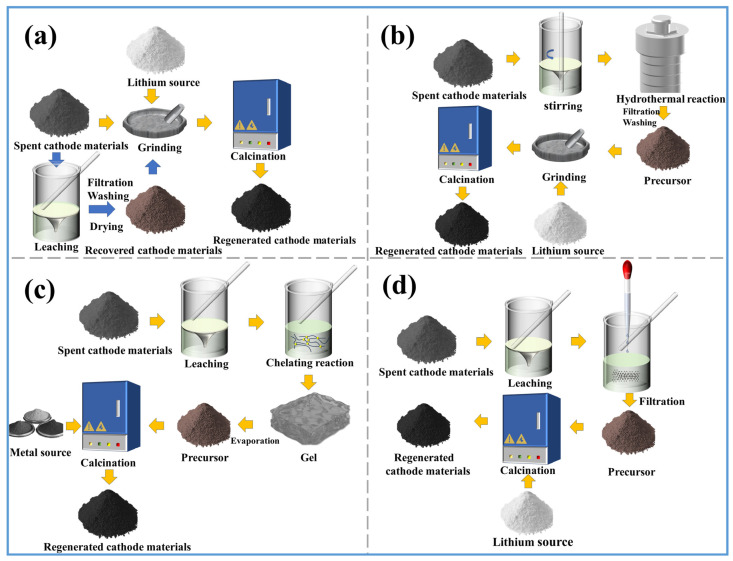
Recycling methods of cathode materials: (**a**) solid-state sintering; (**b**) hydrothermal method; (**c**) sol-gel method; (**d**) co-precipitation method.

Compared to traditional selective precipitation methods, which have complex recovery routes and long separation cycles, hydrometallurgical regeneration can directly synthesize cathode materials or precursors from the leachate. This greatly improves the utilization of electrode materials and achieves closed-loop recycling of spent LIBs. The regenerated cathode can be divided into in-situ lithium replenishment regeneration or re-synthesis regeneration. In-situ lithium replenishment regeneration involves reacting lithium salts such as Li_2_CO_3_ with degraded cathode powder to restore the morphology and electrochemical performance of the cathode material [199]. Re-synthesis regeneration involves mixing extracted metals with related metal salts or metal oxide precursors to produce new cathode materials. The regenerated cathode materials, which fully utilize the recovered valuable metal elements, do not show significant performance degradation and still meet the energy density and cycling performance requirements of LIBs. Regenerated cathode materials usually need to be assembled together with other battery parts to form button batteries to facilitate electrochemical performance testing. The shape of the button battery is shown in Figure 2d. Common regeneration techniques include molten salt synthesis, solid-state sintering, hydrothermal method, sol-gel method, and co-precipitation method, as shown in Figure 13 (the molten salt synthesis and solid-state sintering processes are similar, so they are not depicted separately).

### 6.1. Molten Salt Synthesis (MSS)

The basic principle of molten salt synthesis (MSS) for regenerating cathode materials is to immerse the used lithium-ion battery cathode materials (mainly nickel–cobalt–manganese cathode materials) in high-temperature molten salt solvents for treatment. The cathode material reacts with the molten salt, causing the lithium ions within to dissolve. At an appropriate temperature, the chemical conditions in the molten salt are adjusted to precipitate the dissolved lithium ions onto the electrode surface or another carrier in a different form. Finally, through specific treatment methods, the precipitated lithium ions are converted into a form that can be used again to manufacture new cathode materials, achieving the regeneration of the cathode materials. Early studies used the molten salt method to synthesize cathode materials [200,201], but not based on regenerating spent cathode materials. In recent years, there has been little research on the regeneration of cathode materials using the molten salt method.

### 6.2. Solid-State Sintering

Solid-state sintering is an effective method for processing and regenerating spent cathode materials. First, the precursors need to be uniformly mixed in a ball mill, followed by a two-stage heat treatment process to regenerate the cathode materials. The first stage involves pre-calcination (250–350 °C) to decompose the precursors while removing gases; the second stage involves calcination at higher temperatures (400–800 °C) to obtain the new cathode materials [202]. This method allows for control over the calcination temperature, which influences particle growth, structure, and discharge capacity of the materials, making it suitable for industrial-scale applications. As mentioned earlier, chemical and thermal reduction (CTR) can be used for metal extraction from spent LIBs, and it can also be applied for the regeneration of cathode materials. Recent research has explored the use of rapid/flash Joule heating for the regeneration of cathode materials [203], details of which can be found later in the text. The conventional process flow of solid-state sintering is shown in Figure 13a.

### 6.3. Hydrothermal Method

The hydrothermal method involves placing the pre-treated cathode materials and a hydrothermal reaction promoter in a sealed container to undergo a hydrothermal reaction at 120–220 °C. This method can alter the lattice structure of the cathode material, rearrange the metal ions, and exchange ions with those in the water, forming new compounds or crystal phases. Subsequently, the regenerated products are washed and separated to remove residual impurities. Finally, the regenerated cathode materials undergo drying and can be reintegrated into the manufacturing of new lithium-ion batteries. Furthermore, an annealing process is typically necessary to enhance crystallinity. Annealing plays a crucial role in the hydrothermal regeneration of lithium battery cathode materials, significantly enhancing the material’s structure and electrochemical performance, thereby improving the overall performance of the regenerated battery. The conventional process flow of the hydrothermal method is shown in Figure 13b. Compared with the traditional hydrothermal method, the microwave hydrothermal method can regenerate the positive electrode at shorter reaction times and lower reaction temperatures [204]. In addition, the powder synthesized by the microwave hydrothermal method has high purity, good crystallinity, and a uniform microstructure. Liu et al. [205] self-heated the LCO particles under microwave irradiation, resulting in a highly efficient thermal field inside and near the LiCoO_2_ particles, which improved the crystallinity of regenerated LiCoO_2_.

### 6.4. Sol-Gel Method

The conventional steps of the sol-gel method involve using organic acids as leaching agents and complexing agents. A hydrolysis polymerization reaction occurs based on the leachate, followed by heating to evaporate the water to obtain a sol. Next, the gel is dried to remove the solvent and then sintered at high temperature to transform it into a solid regenerated cathode material. Finally, through processes such as grinding and sieving, the regenerated cathode material is powdered, making it suitable for reuse in the production of new lithium-ion batteries [206,207]. The conventional process flow of the sol-gel method is shown in Figure 13c.

### 6.5. Co-Precipitation Method

The co-precipitation method is commonly used for regenerating ternary cathode materials or processing mixtures of different cathode materials. The conventional steps of this method are (1) acid leaching the pre-treated sample to convert it into a solution. (2) Removing impurity ions from the solution through precipitation or extraction to ensure material purity in subsequent steps. After removing impurities, the ratio of metal ions in the solution is adjusted by adding an appropriate amount of metal salts to achieve the desired chemical composition. (3) Adding a precipitating agent to induce co-precipitation of the metal ions, forming a ternary precursor. (4) Mixing the obtained precursor with the required metal salts and regenerating it through solid-state sintering to convert it into new cathode materials. Commonly used precipitating agents include hydroxides [208], carbonates [209], and oxalates [210]. The conventional process flow of the co-precipitation method is shown in Figure 13d.

### 6.6. Regeneration Process of Nickel–Cobalt–Manganese Cathodes

**Figure 14:**
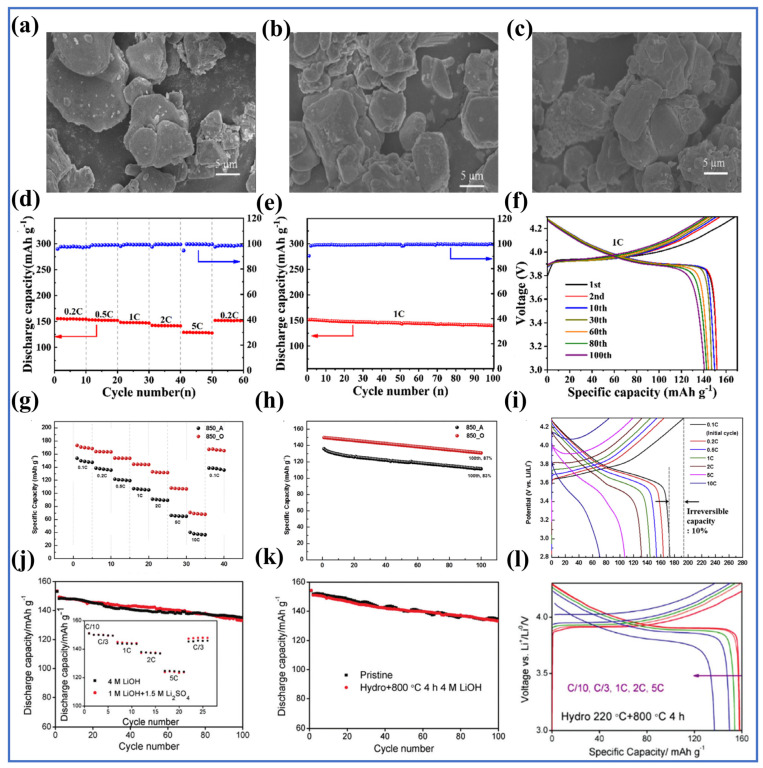
(**a**) Unregenerated LCO morphology, (**b**) morphology of LCO regenerated at 800 °C, (**c**) morphology of LCO regenerated at 900 °C [199], ROYAL SOC OF CHEM; (**d**) rate performance; (**e**) cycling performance at 1C; (**f**) charge–discharge curves at 1C [211], Elsevier; (**g**) rate performance of NCM622 sintered in air and oxygen atmospheres; (**h**) cycling performance at 1C of NCM622 sintered in air and oxygen atmospheres; (**i**) charge–Discharge Curves of NCM622 sintered in oxygen atmosphere [207], Elsevier; (**j**) C cycling and rate performance of recycled LiCoO_2_ treated with pure LiOH and mixed Li salts; (**k**) cycling performance of original and hydrothermally regenerated LCO powders; (**l**) voltage–capacity plots of cathode regenerated by hydrothermal treatment at 220 °C followed by short annealing at 800 °C [226], ROYAL SOC OF CHEM.

Solid-state sintering in the solid-phase reaction method is commonly used for the regeneration of NCM cathode materials, and there has been considerable research on solid-state sintering in recent years. Nie et al. [199] combined LCO cathode powder from dismantled spent LIBs with Li_2_CO_3_ powder (with a Li/Co molar ratio of 1.05), subjected it to air calcination at varying temperatures for 12 h, followed by ball milling and sieving through a 400-mesh sieve to produce regenerated LiCoO_2_. The electrochemical performance enhancement of the regenerated LiCoO_2_ is attributed to the restored layered structure, with markedly improved morphology observed in the regenerated cathode material (Figure 14a–c), particularly optimal at 900 °C. In a simple coin cell assembly, the discharge capacity of the regenerated cathode material reached around 152.4 mAh/g, exhibiting a cycle capacity decay rate of merely 0.0313 mAh/g per cycle. This study pertains to direct in-situ lithium supplementation regeneration. In addition to in-situ regeneration, related metal products extracted through pyrometallurgy can also be used to synthesize cathode materials again. Tang et al. [211] blended the processed cathode material with different proportions of graphite from the anode, then selectively transformed LiCoO_2_ into Co or CoO and Li_2_CO_3_ through carbothermal reduction under vacuum conditions at temperatures ranging from 873 K to 1273 K. The obtained CoO and Li_2_CO_3_ were then mixed and calcined at 750 °C to prepare new cathode materials. The specific capacity of the prepared material was 145 mAh/g, and it retained 93% of its initial capacity after 100 cycles. Detailed rate and cycle performance are shown in Figure 14d–f. Although this secondary synthesis method is more complex than direct in-situ regeneration, the second reaction process allows control over the ratio of Li to transition metals (Ni, Co, Mn), resulting in superior electrochemical performance of the regenerated cathode material. Lin et al. [212] also demonstrated the importance of the Li/transition metal ratio in the performance of regenerated materials. Surface modification of the regenerated cathode can also improve the performance of the regenerated materials. Gao et al. [213] mixed Li_2_CO_3_ with recycled LCO powder and calcined it at 800 °C for 8 h to obtain new LCO cathode powder. They then mixed Al_2_O_3_ as a coating material with the regenerated LCO, ball-milled it at 250 r/min for one cycle, and sintered it at 800 °C in the air for 5 h. After cooling, Al_2_O_3_-coated regenerated LCO was obtained. Electrochemical tests showed that the discharge capacity of the coated material reached 136.8 mAh/g compared to 132.5 mAh/g for uncoated regenerated LCO, indicating capacity improvement. Both regenerated LCO and Al_2_O_3_-coated regenerated LCO exhibited attractive cycle life, retaining 90.1% and 90.2% of their specific capacity after 100 cycles, respectively. While this method enhanced the electrochemical performance of the regenerated material, the degree of enhancement was not significant. Coating the material’s surface consumes high energy, limiting the industrial prospects of this method.

In terms of the molten salt method, Jiang et al. [214] mixed LiOH, Li_2_CO_3_, and pretreated NCM cathode material powder, and sent it to a calcination furnace for two-stage calcination: first at 440 °C for 5 h, and then at 850 °C for 12 h. In the first calcination stage, eutectic molten salt was obtained, achieving lithium replenishment. The spent NCM 523 cathode material was successfully regenerated through lithium eutectic molten salt lithiation and high-temperature calcination. This process restored the original crystal structure, transforming the rock salt phase completely into a layered structure. The regenerated NCM cathode material exhibited excellent cycling stability and high-rate performance, retaining 89.06% of its discharge capacity after 200 cycles at 1C. Although the molten salt method for recovering NCM cathode materials does not require mechanical mixing or multi-stage grinding and heating reagents, the need to prepare molten salt during the calcination process significantly increases the energy consumption of the experiment. Therefore, it cannot replace solid-state sintering as the mainstream pyrometallurgical regeneration process.

Each of the aforementioned methods has its advantages and disadvantages, but they all require prolonged processing at high temperatures to treat spent cathode materials. Recent studies have shown that ultrafast, controllable, and energy-efficient electric heating can be used for material synthesis and processing [215,216]. Yin et al. [203] proposed a rapid Joule heating method for regenerating LCO cathode materials, achieving relithiation and crystal structure repair simultaneously. Following an 8-s repair process, the regenerated LiCoO_2_ displayed a distinct layered structure and reinstated its original electrochemical performance, boasting an initial discharge capacity of 133.0 mAh/g and robust cycling performance. This study achieved cathode material regeneration in a very short time without the addition of other additives, making it more energy efficient and efficient than traditional solid-state reaction methods. Although using rapid/flash Joule heating to regenerate spent lithium battery cathodes is an emerging technology, there is limited research on it, and the electrochemical performance of the regenerated cathodes is relatively low. More research is needed to prove its feasibility. Currently, more research is focused on using rapid/flash Joule heating for the regeneration of lithium battery anodes [217,218].

Before the sol-gel and co-precipitation methods, acid leaching is used to extract the relevant metal ions from the cathode material. In the sol-gel method, to improve the performance of the regenerated cathode material, factors such as the leaching agent, chelating agent, and pre-regeneration calcination temperature need to be considered. Li et al. [206] compared the performance of NCM622 cathode material regenerated using citric acid, glucose, and sucrose as gel chelating agents after acid leaching. The results showed that when glucose was used as the gel, the hexagonal crystal structure was the most complete, the cation mixing was minimal, the impedance was the lowest, and the redox reaction reversibility was the best. To simplify the reaction process and save reagents, Yao et al. [219] used DL-malic acid as a dual-function reagent, serving as both a leaching agent and a chelating agent, successfully regenerating NCM333 cathode material via the sol-gel method. Electrochemical performance tests revealed that the initial charge–discharge capacities of the regenerated cathode material at a 0.2C rate were 152.9 mAh/g and 147.2 mAh/g, respectively, and the capacity retention rate at the 100th cycle was 95.06% of the initial value. Compared to traditional sol-gel methods, this recovery process avoids complex separation of metal ions and generates minimal pollution and by-products. The final step of the sol-gel method requires calcining the gel doped with relevant metal sources, and the calcination temperature affects the regeneration performance. Lee et al. [207] conducted a detailed study on the calcination conditions of sol-gel precursors, focusing on the impact of calcination temperature and atmosphere on the structure and electrochemical performance of NCM622. The study found that at a calcination temperature of 850 °C, the samples exhibited a better-ordered layered structure and higher crystallinity. Additionally, heat treatment in an oxygen atmosphere improved the uniformity of the oxidation state of Ni^2+^ between the surface and interior of NCM622, and suppressed the formation of surface LiOH and Li_2_CO_3_, thus enhancing electrochemical performance. As shown in Figure 14g–i, the NCM622 regenerated through calcination at 850 °C in an oxygen atmosphere achieved a discharge capacity of 174 mAh/g, and after 100 cycles, the capacity retention rate was 89%.

The co-precipitation method is generally used for ternary cathode materials or mixtures of different cathode materials [220]. Apart from the gel complexation step, co-precipitation shares similar procedures with the sol-gel method. Metal ions like Ni, Co, and Mn can precipitate as oxalates, carbonates, or hydroxides. Hydroxide co-precipitation is widely regarded as the predominant method for NCM material preparation [221]. Yang et al. [222] successfully synthesized lithium-ion battery cathode material NCM333 using mixed waste alkaline zinc-manganese cathode and spent cathode as raw materials, dissolved in nitric acid and co-precipitated with sodium hydroxide. The results showed that the regenerated cathode material could provide a capacity of 160.2 mAh/g at 0.1C rate. During precipitation, transition metal hydroxides undergo oxidation in aqueous solutions; for instance, Mn(OH)_2_ gradually oxidizes to MnO_2_ (Mn^4+^) or MnOOH (Mn_3_^+^) under appropriate conditions, leading to reduced uniformity of the final product. To prevent oxidation, the reaction should occur in a closed container under inert gas protection. Conversely, when employing carbonate precipitation, oxidation is not a concern. He et al. [223] synthesized ternary precursors using Na_2_CO_3_ as a precipitant, and the addition of CO_3_^2−^ does not easily change the oxidation state of metal ions, retaining the chemical valence of Mn^2+^, ultimately regenerating NCM333 cathode material with an ordered layered structure and excellent electrochemical performance. This precipitation method also presents drawbacks, including the potential segregation of components during preparation, leading to inadequate assurance of uniform oxide element distribution, thereby adversely impacting the electrochemical performance of NCM materials. Conversely, in the oxalate co-precipitation method, Mn ions maintain stability in an aqueous solution and retain a valence state of +2. Gao et al. [224] dissolved spent NCM523 active material in H_3_PO_4_ and citric acid, adding metal sources as needed to adjust the molar ratio of Ni, Co, and Mn in the leachate to 5:2:3. Then, using oxalic acid precipitation and ammonia to adjust pH, they obtained a precursor, which was then re-sintered and re-lithiated to synthesize NCM523. The initial discharge capacity at 0.2C stood at 149.528 mAh/g, while at 1C, it measured 135.351 mAh/g. Following 100 cycles, the capacity retention rate reached 85.45%. Compared to alternative precipitation methods, oxalate precipitation offers a straightforward synthesis process, stoichiometric component precipitation in solution, and facilitates molecular or atomic-level homogeneous material mixing. Similar to the sol-gel method, the temperature during sintering regeneration is also very important. Chen et al. [218] studied the impact of temperature on the physical and electrochemical properties of regenerated materials by varying the sintering temperature. They found that NCM523 regenerated at 830 °C could still provide a capacity of 149.2 mAh/g after 100 cycles at a 0.2C rate, confirming that optimizing the sintering temperature is crucial for the regeneration of spent ternary cathode materials.

Before hydrothermal treatment, it is usually necessary to calculate the residual lithium content in the cathode material [225,226,227]. After preliminary calculations, Shi et al. [224] placed LiCoO_2_ powder from cycled batteries into a 100 mL Teflon-lined autoclave with either 80 mL of 4 M lithium hydroxide (LiOH) solution or a mixed solution of 1 M LiOH and 1.5 M Li_2_SO_4_. They annealed it at various temperatures, with a control group undergoing regeneration through solid-state sintering. The reaction occurring in the hydrothermal method for LCO can be represented by Equation (56):


(56) $$Li_{x}CoO_{2}+(1-x)LiOH+\frac{x-1}{4}O_{2}\to LiCoO_{2}+\frac{1-x}{2}H_{2}O$$

Samples treated hydrothermally initially showed poor cycling stability, even worse than untreated materials. After annealing at 700 °C for 4 h, stability slightly improved. However, annealing at 800 °C for the same duration significantly enhanced cycling performance. The initial discharge capacity at C/10 was 153.1 mAh/g, dropping to 148.2 mAh/g at 1C, with a retention rate of 91.2% after 100 cycles. The electrochemical performance of the regenerated material is shown in Figure 14j–l. Compared to other regeneration methods, the cathode material prepared by the hydrothermal method exhibited better rate performance due to smaller charge transfer resistance and a higher Li^+^ diffusion coefficient. In subsequent research, they applied the same method to regenerate NCM cathode materials, achieving ideal stoichiometry, minimal cation mixing, and high phase purity. The regenerated materials showed high specific capacity, excellent cycling stability, and superior rate performance [227].

**Figure 15:**
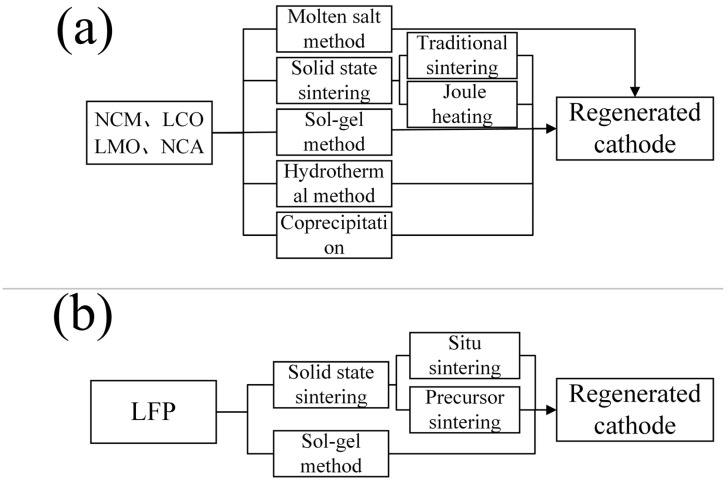
(**a**) Regeneration process of nickel–cobalt–manganese cathodes; (**b**) regeneration process of LFP cathode.

Overall, the sol-gel method has advantages in controlling performance, the co-precipitation method is relatively simple but requires attention to reaction conditions, and the hydrothermal method can improve the cycling stability and rate performance of materials. Although wet regeneration technologies may have certain advantages in laboratory research, solid-state sintering is still the mainstream choice in actual industrial production. The regeneration process for nickel–cobalt–manganese cathode materials can be referred to in Figure 15a.

### 6.7. Regeneration Process of LFP Cathode

**Figure 16:**
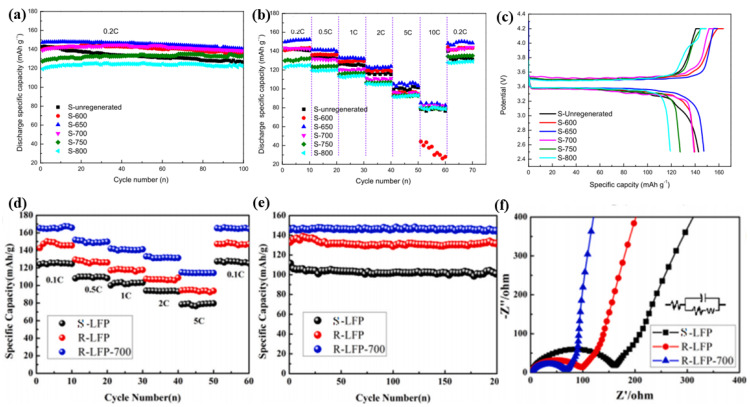
Cycling performance (**a**) and rate performance (**b**) curves of regenerated cathode material mixtures at different temperatures; (**c**) first cycle charge–discharge curves of the regenerated cathode material mixtures at different temperatures [230], Elsevier; (**d**) cycling performance of S-LFP, R-LFP, and R-LFP-700 at different rates; (**e**) cycling performance of S-LFP, R-LFP, and R-LFP-700 at 1C rate; (**f**) electrochemical impedance spectroscopy of S-LFP, R-LFP, and R-LFP-700 [235], Elsevier.

When using solid-state sintering to regenerate LFP, the temperature should not exceed 800 °C. Beyond 800 °C, the incomplete decomposition of PVDF during separation produces HF, which reacts with Li in LFP to form LiF, and LiFeO_4_ also decomposes, severely affecting the electrochemical performance of the new cathode [228]. Song [228] subjected pretreated LFP powder to solid-phase sintering at 600 °C, 700 °C, and 800 °C in an inert atmosphere and set up control groups with and without doping of new LFP cathode powder. The results showed that at 700 °C, the highest cathode capacity at a 1C rate was 144 mAh/g. Regenerated samples without new LiFePO_4_ doping could not meet the reuse requirements due to a low initial capacity of 102 mAh/g. This study indicates that direct solid-state sintering of spent LFP alone is insufficient to regenerate a new cathode. Subsequent research often involves solid-state sintering of LFP with retained acetylene black. For example, Wang et al. [229] retained acetylene black in the cathode during separation and sintered the resulting cathode material mixture at 750 °C in an N_2_ atmosphere for 7 h to obtain regenerated cathode material. The regenerated cathode material mixture displayed superior electrochemical performance compared to recycled materials, boasting an initial discharge capacity of 129.43 mAh/g. Even after 1000 cycles at 0.5C in an 18,650 battery test, it retained a capacity of 120.32 mAh/g, demonstrating a high retention rate of 92.96%. However, the study highlighted the necessity for a relatively long regeneration time despite significantly enhancing the electrochemical performance of the regenerated cathode. Li et al. [230] performed solid-state sintering on the separated “LiFePO_4_ + acetylene black” doped with Li_2_CO_3_, achieving a rapid 1-h regeneration of LFP. The cycling performance and charge–discharge curves of the regenerated cathode at different temperatures are shown in Figure 16a–c. The LFP regenerated at the optimal temperature (650 °C) exhibited a maximum discharge specific capacity and coulombic efficiency of 147.3 mAh/g and 92.96% at a 1C rate. After 100 cycles, the discharge-specific capacity and capacity retention rate remained at 140.4 mAh/g and 95.32%. This method ensured excellent electrochemical performance while achieving rapid regeneration. Besides the direct in-situ solid-state sintering regeneration of spent LFP, another method involves extracting Li compounds and FePO4 via the wet process mentioned in Section 5.4, followed by their solid-state sintering. For instance, Jin et al. [184] obtained regenerated LFP by solid-state sintering a mixture of Li_2_CO_3_ and FePO_4_ recovered through acid leaching with glucose. The specific capacities of the regenerated material at 0.2, 0.5, 1, 2, and 0.2C (1C = 170 mAh/g) were 148.8, 142, 131, 114.5, and 149.0 mAh/g, respectively. Such studies are common steps in the wet recovery of LFP and effectively utilize the relevant elements from spent LFP. However, due to the need to control multiple material ratios during the experimental process, the industrial prospects are less favorable compared to in-situ LFP sintering regeneration.

Although solid-state sintering is highly regarded for its practicality and simplicity, this method of solid-phase calcination also has some notable drawbacks: firstly, during high-temperature sintering, the lithium source does not uniformly contact the spent LFP; secondly, the pyrolysis of binders and residual electrolytes releases toxic gases that pollute the environment [231,232]. In contrast, during hydrothermal regeneration of LFP, soluble Li+ ions can freely diffuse in the aqueous solution, ensuring direct contact with FP during regeneration and avoiding non-uniform contact issues. Jing et al. [233] utilized a hydrothermal regeneration system with Li_2_SO_4_·H_2_O providing Li+ ions, while N_2_H_4_·H_2_O served as a reducing agent to maintain the solution’s low redox potential. The regenerated LFP displayed outstanding discharge capacities of 146.2 mAh/g at 0.2 C, 141.9 mAh/g at 1 C, and 128.2 mAh/g at 5 C. After 200 cycles at 1 C, the capacity retention rate reached 98.6%. Despite the excellent electrochemical performance observed, the use of N_2_H_4_·H_2_O as a reducing agent raises regeneration costs due to its relative expense. Yang et al. [234] used the inexpensive Na_2_SO_3_ as a reducing agent and Li_2_SO_4_ solution as the lithium source to directly regenerate spent LFP through a 6-h hydrothermal reaction. They systematically studied the effects of reducing agent amount, lithium-ion concentration, and hydrothermal temperature on LFP regeneration. In the hydrothermal reduction system, the spent LFP cathode material’s chemical structure and electrochemical performance were successfully restored within 6 h at 200 °C. Regenerated LFP exhibited excellent electrochemical performance, with specific capacities at various rates. After 100 cycles at 1C, the capacity retention rate exceeded 99%. Additionally, after 100 cycles at 1C, the capacity retention rate was >99%. It is worth mentioning that although Na_2_SO_3_ is cheaper than N_2_H_4_·H_2_O, the hydrothermal reaction time is longer when using Na_2_SO_3_, so it does not necessarily mean that Na_2_SO_3_ is more economical than N_2_H_4_·H_2_O. Adding a calcination process after the hydrothermal method can significantly enhance the performance of regenerated LFP. Chen et al. [235] used LiOH·H_2_O as the lithium source and tartaric acid as the reducing agent to react with spent LFP hydrothermally at 200 °C for 2 h, then calcined the reacted materials in a tube furnace at 700 °C for 3 h. Compared to the non-calcined samples, the regenerated LFP showed significantly improved electrochemical performance (Figure 16d–f), with specific capacities of 165.9, 151.93, 145.92, 133.11, and 114.96 mAh/g at 0.1, 0.5, 1, 2, and 5C, respectively. Additionally, after 200 cycles at 1C, the capacity retention rate was as high as 99.1%.

Overall, hydrothermal regeneration is more suitable for the industrial regeneration of LFP batteries due to its better uniformity and environmental friendliness, but it requires more precise control of conditions; the solid-state sintering method is simple and can be deployed quickly, but it suffers from high-temperature pollution and uneven product issues. To achieve industrial production, combining the two methods to leverage their respective strengths could be considered. For example, using hydrothermal treatment for preliminary regeneration to ensure product uniformity and environmental protection, followed by a short-duration solid-state calcination at a lower temperature to enhance the material’s electrochemical performance and production efficiency. This combined approach might achieve a better balance and effectiveness in industrial applications. The regeneration process used for the LFP cathode material can be referred to in Figure 15b.

## 7. Recommendations and Outputs 

The recycling technologies for spent LIBs have made significant advancements, yet there remains ample room for further development. This paper synthesizes the existing spent LIB recovery technology and existing problems, and puts forward some suggestions on this basis. 

(1) The pretreatment of spent LIBs is a critical step to ensure the efficiency, safety, and environmental friendliness of the recycling process. Current pretreatment technologies have reached a certain scale but still have significant room for improvement. In the future, innovation and optimization in areas such as automation, intelligence, green technologies, efficient energy management, integrated recycling systems, standardization, and regulation can further enhance the efficiency and effectiveness of pretreating spent LIBs. Discovering a universal disassembly route suitable for all batteries could significantly enhance the efficiency of industrial lithium battery recycling.

(2) High-temperature smelting for recycling spent LIBs offers efficient and high-purity metal recovery with mature processes capable of handling complex materials. However, its high energy consumption, pollution, and costs limit its widespread use. Similarly, the carbon thermal reduction (CTR) method is efficient and cost-effective for large-scale metal recovery but faces challenges with high-temperature operations and carbon emissions. The salt-assisted roasting method optimizes recovery rates and lowers reaction temperatures but must address salt recovery and equipment corrosion issues. Future research for pyrometallurgy methods should focus on reducing energy consumption and pollution while improving recovery rates, especially as environmental regulations tighten and technology advances.

(3) Acid leaching is advantageous for its rapid reaction and high efficiency, but it is highly corrosive and requires treatment of hazardous waste liquids. Ammonia leaching is environmentally friendly with high selectivity, yet it involves a complex process and long reaction times. Bioleaching is environmentally friendly with low energy consumption, but it has long recovery cycles and lower efficiency. DESs have lower toxicity and are recyclable, but their technology is still under development and they are relatively costly. Currently, acid leaching remains the mainstream wet leaching method; however, in the future, DES leaching may have broader applications due to its environmental friendliness and sustainability.

(4) Currently, chemical precipitation and organic solvent extraction are widely used due to their lower initial costs and established processes. However, the future trend is shifting towards more sustainable and high-purity recovery methods. The integration of electrochemical and ion exchange technologies is seen as a key future direction due to their potential for high selectivity, reduced environmental impact, and suitability for circular economy models.

(5) Compared to pyrometallurgy and hydrometallurgy, the regeneration method is the most resource-efficient approach. The recovered materials can be directly used to manufacture new batteries, reducing complex post-treatment steps such as metal purification and remanufacturing, thus enhancing the market competitiveness of the products. In the regeneration method, solid-state sintering technology offers fast recovery with fewer steps, making it more suitable for industrial recycling. 

(6) The recycling market for spent lithium batteries has not yet been fully developed, and the market mechanisms are incomplete, leading to certain difficulties for recycling companies. The high costs of equipment, technology, and labor in the recycling process result in limited economic benefits. Especially with the significant fluctuations in metal prices, the economic viability of recycling is challenged. 

(7) There are difficulties in regulation and enforcement during the recycling process, especially in the collection and transportation of spent batteries, where violations and illegal dumping occur. Although some countries and regions have introduced relevant policies, overall policy support is insufficient, failing to form a systematic and standardized recycling management system. More policy support is needed for the spent lithium battery recycling industry. 

(8) This paper reviews the research on the cathode materials of spent lithium batteries, but the anode and electrolyte of lithium batteries also have potential for recycling. Currently, there is limited research on the recycling of anodes and electrolytes. Continuous technological innovation and process optimization are needed to solve the efficient recycling of anodes and electrolytes, achieving a complete and systematic recycling process for spent lithium batteries.

## 8. Conclusions

In recent years, the electric vehicle (EV) has developed rapidly, with LIBs playing a crucial role as the core component. As EVs become more widely adopted, the number of LIBs reaching the end of their lifespan is increasing significantly. A similar issue is seen with mobile phones and other electronic devices, whose widespread use has also led to a substantial increase in lithium battery consumption. Given that these devices typically have shorter lifespans, a large number of lithium batteries are being retired sooner. Retired lithium batteries contain a large amount of harmful substances, and improper disposal can cause severe environmental pollution. Additionally, lithium batteries contain valuable rare metals such as lithium, cobalt, and nickel, which have high recycling value. Therefore, establishing a comprehensive recycling system for spent lithium batteries not only effectively reduces environmental pollution but also enables resource reuse, promoting a sustainable circular economy. To address the disposal issues of spent LIBs, extensive research has been conducted both domestically and internationally. This paper first introduces the structure and working principles of lithium batteries, as well as the pretreatment steps required for cathode recovery. Then, it summarizes the mechanisms and processes of recent pyrometallurgical and hydrometallurgical methods for extracting valuable metals and provides a detailed explanation of the methods for regenerating cathode materials. Based on this, the paper presents a detailed analysis and comparison of the recycling processes, products, and efficiencies of nickel–cobalt–manganese cathodes (NCM/LCO/LMO/NCA) and lithium iron phosphate (LFP) cathodes. Finally, we summarize the current shortcomings in the spent LIB recycling industry, evaluated the advantages and disadvantages of various recycling technologies, and provided recommendations for the future development of the recycling industry, including the necessary policy support. Overall, the recycling and reuse of metal resources such as lithium, cobalt, and nickel from lithium batteries can not only reduce dependence on primary mineral resources but also effectively alleviate resource shortages, promoting the circular utilization of resources and sustainable development.

## Figures and Tables

**Table 1:** Characteristics and latest developments of common batteries [6,7,8,9,10].

Battery Type	Energy Density (Wh/kg)	Common Working Voltage (V)	Cycle Life (Times)	Cost	Environmental Impact	Recent Development
Alkaline	50–150	1.5	N/A	Low	Moderate	Alkaline zinc-based batteries (widely used in daily energy storage)
Nickel–Cadmium	45–80	1.2	500–1500	Moderate	High (toxic metals)	N/A
Nickel–Metal Hydride	60–120	1.2	500–1000	Moderate	Lower than NiCd	Nickel hydrogen gas batteries (applied to aerospace and power grid scale)
Lead-Acid	50–100	2	500–2500	Low	High (toxic lead)	Use as power batteries (risk of being replaced)
Lithium-Ion	110–160	2.5/3.3/3.6–3.7	1000–8000	High	Lower than NiCd and Lead-Acid	Solid-state batteries, silicon anode research, improved safety features

**Table 2:** Contents and properties of each composition in LIBs [34,35,36,37,38,39].

Component	Content by Weight (wt%)	Composition	Structure	Characteristics/Advantages
Cathode	39.10 ± 1.1	LCO	Layered	High structural stability, over 500 cycles, 80–90% capacity retention
		LMO	Spinel	Economical, environmentally friendly
		NCA	Layered	Higher energy density, but lower structural stability
		LFP	Olivine	Stable structure, low cost
		NCM	Layered/Spinel	High capacity, good thermal stability
Anode		Carbon	Graphite	Low cost, abundant resources, high reversibility
Electrolyte		LiPF_6_, LiClO_4_, LiBF_4_	Liquid	High conductivity, wide operating temperature range
Separator	5.20 ± 0.4	PE/PP	Polyolefin	Porous, allows ion flow, prevents short circuits
Plastic shell	22.90 ± 0.7	Carbonized plastic layer		Sealed battery
Other shells	10.50 ± 1.1	Of stainless steel, aluminum		
Copper foil	8.90 ± 0.3	Copper		Thickness ~14 µm
Aluminum foil	6.10 ± 0.6	Aluminum		Thickness ~20 µm
Binder	2.00 ± 0.5	PVDF, CMC, SBR		Binds electrode materials

## Data Availability

Not applicable.

## List of Abbreviations

LIBs	Lithium-ion batteries	CMC	carboxymethyl cellulose
NCM	Lithium Nickel Cobalt Manganese Oxide	EC	ethylene carbonate
LMO	Lithium Manganese Oxide	PC	propylene carbonate
LFP	Lithium iron phosphate	DMC	dimethyl carbonate
LCO	Lithium Cobalt Oxide	MAPC	methyl acrylate carbonate
NCA	Lithium nickel cobalt aluminum oxide	DME	dimethoxyethane
EV	Electric vehicle	PE	polyethylene
BEV	Battery electric vehicle	PP	polypropylene
PHEV	Plug-in hybrid electric vehicle	TOA	trioctylamine
PVDF	polyvinylidene fluoride	TBP	Tri-Butyl Phosphate
PTFE	polytetrafluoroethylene	D2EHPA	Di-(2-ethylhexyl) phosphoric acid
DESs	Deep eutectic solvents	CTR	Carbon thermal reduction
ILS	Ionic liquids	HTR	Hydrogen thermal reduction
